# Clarification on the Reactivity of Diaryl Diselenides toward Hexacyclohexyldilead under Light

**DOI:** 10.3390/molecules26206265

**Published:** 2021-10-16

**Authors:** Vu Thai Hung, Cong Chi Tran, Yuki Yamamoto, Shintaro Kodama, Akihiro Nomoto, Akiya Ogawa

**Affiliations:** Department of Applied Chemistry, Graduate School of Engineering, Osaka Prefecture University, Osaka 599-8531, Japan; vth261@gmail.com (V.T.H.); mz105131@edu.osakafu-u.ac.jp (C.C.T.); syb02137@edu.osakafu-u.ac.jp (Y.Y.); nomoto@chem.osakafu-u.ac.jp (A.N.)

**Keywords:** diaryl dichalcogenides, hexacyclohexyldilead, photoirradiation, homolytic substitution

## Abstract

In this study, the reactivity of organochalcogen compounds toward a representative alkyl-lead bond compound under light was investigated in detail. Under light irradiation, the Cy-Pb bond of Cy_6_Pb_2_ (Cy = cyclohexyl) undergoes homolytic cleavage to generate a cyclohexyl radical (Cy•). This radical can be successfully captured by diphenyl diselenide, which exhibits excellent carbon-radical-capturing ability. In the case of (PhS)_2_ and (PhTe)_2_, the yields of the corresponding cyclohexyl sulfides and tellurides were lower than that of (PhSe)_2_. This probably occurred due to the low carbon-radical-capturing ability of (PhS)_2_ and the high photosensitivity of the cyclohexyl-tellurium bond.

## 1. Introduction

Organochalcogen compounds are widely used as functional materials and pharmaceuticals [1,2]. These functional molecules are mainly synthesized using ionic and metal-assisted reactions [3,4,5,6,7,8,9,10,11,12,13,14,15,16,17]. To develop new functional molecules that will support future society, conventional synthetic methods alone are insufficient. Therefore, the development of innovative molecular transformation methods based on the elucidation of unexplored elemental properties is essential. By focusing on the radical reaction properties of organochalcogen compounds, we successfully developed a series of new addition reactions based on radical mechanisms [18,19,20,21,22,23,24]. These reactions can be induced by light irradiation and exhibit high functional group selectivity, which is a characteristic of radical reactions. During the course of this study, we found that selenium and tellurium compounds have excellent carbon-radical-trapping abilities. This observation prompted us to investigate the radical reactions of heavier elements in groups 14 and 15. For instance, triarylbismuthine (Ar_3_Bi) can generate aryl radicals upon photoirradiation. Moreover, we conveniently synthesized aromatic monoselenides by trapping the generated aryl radicals with diselenides [25]. To further elucidate the photoreactivity of organic dichalcogenides toward the bonds between heavier elements and alkyl carbons, we next investigated the photoinduced reactions of organic dichalcogenides with alkyl-heavier-element compounds. Most compounds containing heavier-element-aliphatic carbon bonds are unstable, and those that can be isolated are limited. In this study, hexacyclohexyldilead (Cy_3_Pb-PbCy_3_) was selected as a model compound, and its reaction with organic dichalcogenides was investigated in detail.

Among the organic compounds containing heavier group elements, the synthetic applications of organolead compounds have been limited due to the widespread belief that their high toxicity causes serious harm to the environment as well as to human health. However, lead does not exhibit intense acute toxicity. The Library of Chemical Safety Data suggests that lead is approximately one-tenth as toxic as palladium [26]. However, chemists should be careful of the chronic toxicity caused by these compounds. Aromatic lead compounds can be used as arylating agents for various organic molecules [26,27,28]. On the contrary, alkyl-lead compounds are very limited, partly because they are less stable than aromatic lead compounds. In most organolead compounds [29,30], the stable valency of Pb is +4. Moreover, the stability of these compounds varies with the type of organic groups. For tetraalkylleads (R_4_Pb), organolead compounds with secondary alkyl groups are typically less stable than the corresponding compounds with primary alkyl groups. This difference in stability is caused by the steric hindrance of the secondary alkyl groups. Organolead compounds with tertiary alkyl groups have not yet been synthesized. These isolable alkyllead compounds are generally insoluble in aqueous solvents. However, they are relatively stable in dilute acids or bases. When dissolved in organic solvents, they often undergo reactions involving carbon–lead bond cleavage. Tetraethyllead is usually added to gasoline as an antiknock agent. As alkyl–lead bonds are generally weak, they undergo homolysis upon photoirradiation or heating. Hence, the generated alkyl radicals contribute to the antiknock effect. Hexaalkyldileads (R_6_Pb_2_), which are less common than tetraalkylleads, generally exist in the form of liquids and are often difficult to synthesize in the pure form. In contrast, hexacyclohexyldilead (**1**, Cy_6_Pb_2_) [31] is an air-stable and nonpyrophoric solid. It was reported that **1** can be prepared by the reaction of PbCl_2_ with cyclohexylmagnesium bromide; moreover, **1** was found to be very sensitive to light. However, only a few studies have discussed the reactivity of **1** under light in detail. Hence, we selected **1** as a model compound and reported the reactions of organic dichalcogenides, such as (PhS)_2_, (PhSe)_2_, and (PhTe)_2_, with hexacyclohexyldilead **1** under light.

## 2. Results and Discussion

Hexacyclohexyldilead (**1**) was synthesized by the reaction of PbCl_2_ with excess amounts of cyclohexylmagnesium bromide in diethyl ether and was isolated as a pale-yellow solid [31].

**Figure 1:**
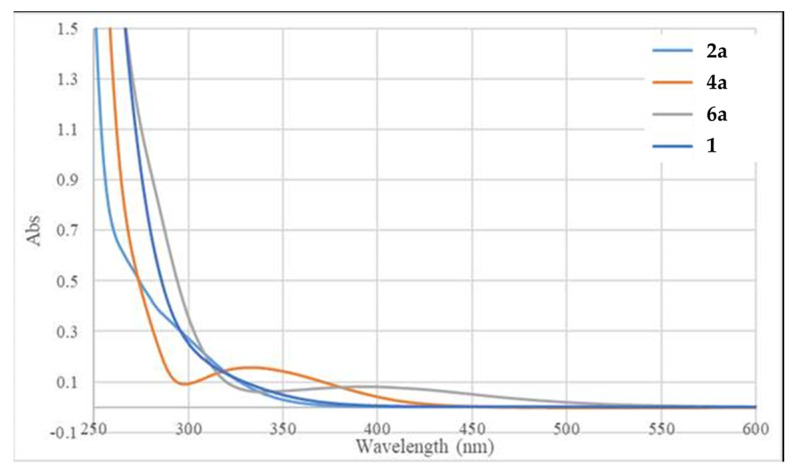
UV–visible spectra of hexacyclohexyldileads **1**, (PhS)_2_ **2a**, (PhSe)_2_ **4a**, and (PhTe)_2_ **6a** (0.02 M in CHCl_3_).

As **1** was reported to be light-sensitive, we first measured its UV–visible spectrum (dark blue line in Figure 1). A dilute solution of **1** (0.02 M in CHCl_3_) exhibited absorption in the UV and near-UV regions, with a cutoff wavelength of 365 nm. Accordingly, upon irradiation with UV or near-UV light, **1** decomposed to generate a cyclohexyl radical.

On the other hand, organic dichalcogenides such as (PhS)_2_ **2a**, (PhSe)_2_ **4a**, and (PhTe)_2_ **6a** exhibited absorption maxima at 250, 340, and 406 nm, respectively. Therefore, when irradiated with light from these regions, the chalcogen-chalcogen single-bond in these compounds underwent homolytic cleavage to generate the corresponding chalcogen-centered radicals. In the absence of substrates, these radicals easily recombine at the rate of diffusion control to re-form the starting dichalcogenides. In addition, the carbon-radical-capturing abilities of (PhS)_2_, (PhSe)_2_, and (PhTe)_2_ were reported to be 7.6 × 10^4^, 1.2 × 10^7^, and 4.8 × 10^7^ Mol^−1^s^−1^, respectively [32]. With these kinetic data in mind, we examined the photoinduced reactions of **1** with diphenyl dichalcogenides such as (PhS)_2_ (**2a**), (PhSe)_2_ (**4a**), and (PhTe)_2_ (**6a**) (Table 1).

The reaction of **1** with **2a** under a white LED lamp through Pyrex for 24 h afforded cyclohexyl phenyl sulfide (**3a**) in 26% yield (entry 1). When a xenon lamp was used as the light source, the yield of **3a** increased (42%) after 4 h of irradiation (entry 2). Upon heating the reaction mixture at 100 °C in toluene in the dark, the reaction of **1** and **2a** barely proceeded (entry 3). In the dark, the addition of benzoyl peroxide caused the formation of **3a**, but the yield was lower (entry 4). Under the same conditions as entry 1, (PhSe)_2_ **4a** reacted more efficiently with **1** to afford the corresponding selenide **5a** in 82% yield (entry 5). Using other light sources, the desired reaction proceeded efficiently to afford **5a** in good yields (entries 6–8). Notably, the use of a xenon lamp successfully resulted in an excellent yield of **5a** (entry 8). Using benzene or acetonitrile as the solvent slightly decreased the yield of **5a** (entries 9–10). CHCl_3_ was found to be the most suitable solvent for this reaction. When MeCN was utilized as the solvent, the yield of **5a** decreased due to the low solubility of **1** in MeCN (entry 10). The reactions of **1** with **4a** afforded higher product yields than those with **2a;** this was due to the higher carbon-radical-capturing ability of **4a** (the rate constants of the S_H_2 reaction of alkyl radicals with **4a** are known to be much higher than those with **2a** by a factor of ca. 160) [32]. The photoinduced reaction of **1** with **6a** afforded the corresponding telluride **7a** (entry 11). The relatively low yield of **7a** can be explained by the instability of **7a** under light. As a result, the conditions described in entry 8 were chosen as the optimized reaction conditions for trapping the cyclohexyl radical with dichalcogenide (i.e., (PhSe)_2_).

**Scheme 1:**
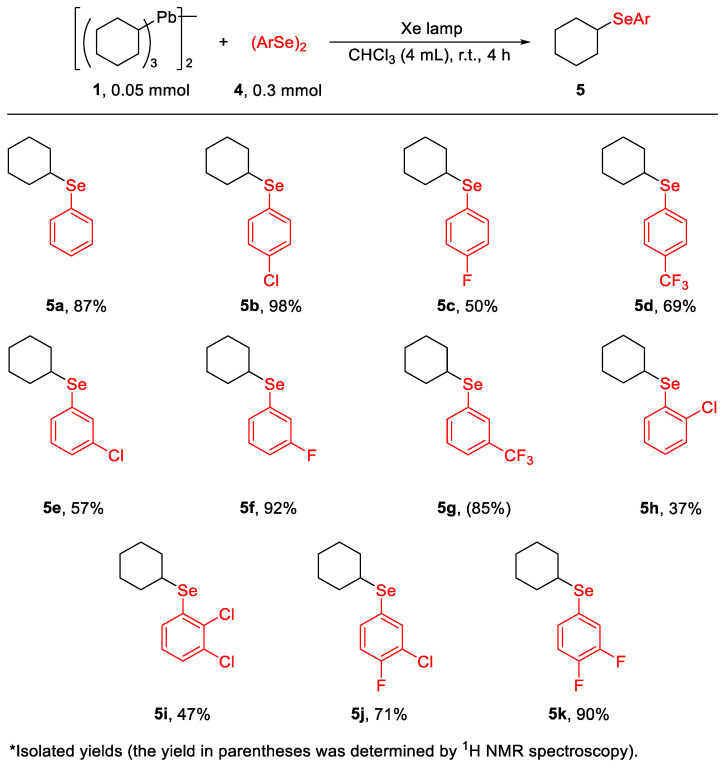
Substrate scope for the reaction of diaryl diselenides (**4**) with **1**.

Next, we examined the substrate scope for the reaction of various diaryl diselenides (**4**) with **1** (Scheme 1). The reaction tolerated a wide range of electron-deficient diselenides (**4b**–**4g**). The reaction with *p*-substituted diaryl diselenides (**4b**–**4d)** afforded selenides **5b**–**5d** in moderate to excellent yields. *m*-Substituted diselenides **4e**–**4g** also afforded aryl cyclohexyl selenides **5e**–**5g** in good yields. In contrast, *o*-substituted diselenides **4h** and **4i** afforded **5h** and **5i**, respectively, in low yields. This probably occurred due to the steric hindrance caused by the *ortho*-substituents. The reactions of **1** with bifunctionalized diselenides **4j** and **4k** also afforded selenides **5j** and **5k**, respectively, in high yields.

During the selenide synthesis, all cyclohexyl groups of **1** were used for the preparation of aryl cyclohexyl selenide **5**. This can be clearly observed from the reaction of **1** with **4b**. Moreover, the presence of near-UV irradiation is the driving force of this reaction. This reaction does not require heating at high temperatures, the use of additives, or long reaction times.

**Scheme 2:**
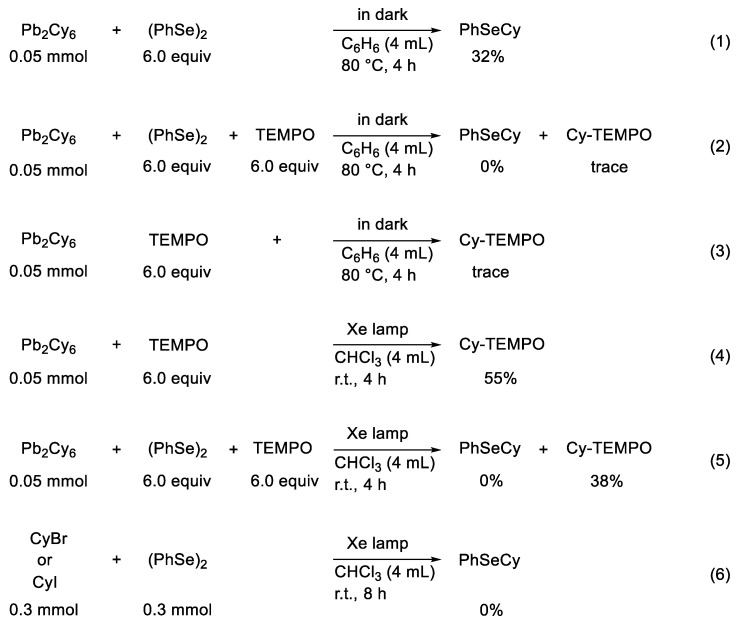
Mechanistic experiments.

Several mechanistic experiments were carried out to understand the reaction pathway as shown in Scheme 2 (Equation (1)–(6)). It was reported that the homolysis of (PhSe)_2_ **4a** occurred upon heating at 80 °C [33]. Hence, we examined the thermal reaction between **4a** and **1**. Under heating conditions, cyclohexyl phenyl selenide (**5a**) was formed in 32% yield (Equation (1)). In addition, **1** was stable at the temperature in the dark (vide post). These results suggest that the thermal reaction might be initiated by the phenylseleno-radical (Equation (1)). Moreover, the addition of a radical-trapping reagent ((2,2,6,6-tetramethylpiperidin-1-yl)oxyl (TEMPO)) prevented the formation of the product (Equation (2)). Therefore, we can conclude that the thermal reaction between **1** and **4a** (Equation (1)) proceeds via a radical pathway. The thermal reaction of **1** with TEMPO suggests that cyclohexyl radicals were not generated by **1** under heating conditions (Equation (3)). Under light, the cyclohexyl radical generated by **1** was successfully trapped by TEMPO, and Cy-TEMPO was formed in good yield as the sole product (Equation (4)). When TEMPO was added to the standard reaction of **4a** and **1**, Cy-TEMPO was formed instead of the selenide adduct (Equation (5)). This result strongly suggests that the formation of aryl cyclohexyl selenide might proceed via a radical reaction pathway. In addition, the reactions of alkyl halides, such as cyclohexyl bromide and iodide, with diphenyl diselenide did not produce **5a** (Equation (6)).

**Scheme 3:**
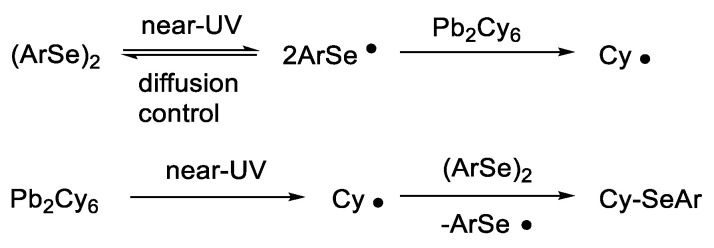
Possible pathways for the formation of aryl cyclohexyl selenide **5**.

Scheme 3 describes possible pathways for the photoinduced reaction of diaryl diselenide **4** with **1**. Irradiation with near-UV light causes the cleavage of the Se-Se bond of **4** and the C-Pb bond of **1**, generating the arylseleno radical (ArSe•) and cyclohexyl radical (Cy•), respectively. Most of the arylseleno radicals easily recombine with each other to re-form (ArSe)_2_ at the rate of diffusion control. It is possible that ArSe• induces the generation of Cy• from **1**; however, this might not be the major reaction in this pathway to afford Cy-SeAr. The Cy• radical undergoes an S_H_2 reaction with **4** to afford aryl cyclohexyl selenide (**5**).

In recent years, several photochemical C-Se bond formation reactions have been reported [34,35,36,37,38,39,40]. The use of photocatalysts is one of the effective methods to achieve this type of transformation under visible light irradiation [34,35]. Photocatalyst-free C-Se bond formation reactions under UVA [38] or visible light [36,37,39] irradiation have also been reported, but an oxidant (O_2_) or a base is often needed. The present reaction provides a new and simple photochemical C-Se bond formation reaction without a photocatalyst or a base.

## 3. Materials and Methods

### 3.1. General Information

Unless otherwise stated, all starting materials were purchased from commercial sources and used without further purification. All solvents were distilled before use. ^1^H NMR spectra were recorded in CDCl_3_ using the JEOL JNM-ECX400 (400 MHz) FT NMR and JEOL JNM-ECS400 (400 MHz) FT NMR systems (Tokyo, Japan) with Me_4_Si as the internal standard. ^13^C{^1^H} NMR spectra were recorded in CDCl_3_ using the JEOL JNM-ECX400 (100 MHz) FT NMR and JEOL JNM-ECS400 (100 MHz) FT NMR systems (Tokyo, Japan) with Me_4_Si as the internal standard. ^19^F{^1^H} NMR spectra were recorded using a JEOL JNM-ECS400 (373 MHz) FT NMR system (Tokyo, Japan) in CDCl_3_ with CFCl_3_ as the internal standard. ^77^Se{^1^H} NMR spectra were recorded on the JEOL JNM-ECX400P (76 MHz) FT NMR system (Tokyo, Japan) in CDCl_3_ with (PhSe)_2_ (463 ppm) as the external standard. IR spectra were reported in wave numbers (cm^−1^). GC–MS spectra were obtained using a Shimadzu GCMS-QP5000 instrument (Kyoto, Japan). High-performance liquid chromatography (HPLC) (recycle GPC) was performed on a Japan Analytical Industry LC-908 (Tokyo, Japan) with JAIGEL-2HH (polystyrene-based column) for isolating the products.

### 3.2. Procedure for Synthesis of Hexacyclohexyldilead ***1***

To a solution of cyclohexylmagnesium bromide, which was prepared by the reaction of cyclohexyl bromide (10 mmol) and magnesium (10 mmol) in diethyl ether (20 mL), PbCl_2_ (5 mmol) and benzene (10 mL) were added. The mixture was then stirred and refluxed at 80 °C for 4 h. After quenching the resulting mixture with 3 M aq. HCl (15 mL) at room temperature, the precipitated black solid was removed by filtration. The resulting filtrate was extracted with Et_2_O (5 mL × 3). Slow evaporation of the organic layers resulted in the precipitation of pale-yellow solids. The product was collected by filtration and washed with isohexane to afford Cy_6_Pb_2_ **1** (1.22 g, 1.3 mmol, 53% yield). ^1^H and ^13^C{^1^H} NMR spectra of **1** are included in the Supplementary Materials.

Hexacyclohexyldilead (**1**) (CAS: 6713-82-2) [41]. Yellow solid, mp >250 °C; ^1^H NMR (400 MHz, CDCl_3_): δ 2.95–2.89 (m, 6H, 6 × C*H*); 2.44–1.32 (m, 60H, 30 × C*H*_2_); ^13^C{^1^H} NMR (100 MHz, CDCl_3_): δ 34.1 (*C*H), 33.7 (*C*H_2_), 30.2 (*C*H_2_), 26.5 (*C*H_2_).

### 3.3. General Procedure for Synthesis of Diaryl Diselenides

Diselenides **4b**–**4k** were prepared according to the literature [42,43]. To a 100 mL three-necked flask, 30 mmol of Mg powder (0.48 g), a stirring bar, and a small piece of I_2_ were added. The flask was then fitted with a condenser and was charged with N_2_. Under N_2_ atmosphere, 10 mL of anhydrous ether was added to the flask. Subsequently, a small amount of aryl bromide was added. After initiation, the rest of the aryl bromide (20 mmol) in anhydrous ether (10 mL) was slowly added to the reaction mixture with ice-water bath cooling. After the preparation of the Grignard reagent, 1.6 g of selenium powder (20 mmol) was added slowly. After stirring for 1 h, the mixture was poured into 40 mL of 2 M HCl solution with ice. The mixture was extracted with ether (50 mL) three times, and the combined organic layer was dehydrated using anhydrous Na_2_SO_4_. The solution was then charged with O_2_ for 24 h. Distillation of the solvent afforded crude diselenide, which was purified by column chromatography (eluent: hexane).

### 3.4. General Procedure for the Reaction of Diaryl Diselenides with Hexacyclohexyldilead

To a sealed Pylex glass tube was added **1** (45.6 mg, 0.05 mmol), **4** (0.3 mmol), and CHCl_3_ (4 mL) under inert atmosphere. The mixture was irradiated using a xenon (500 W) lamp from a distance of 15 cm. After 4 h, the reaction mixture was run through a short bed of silica gel with EtOAc as the eluent. After the evaporation of the solvent, product **5** was obtained by gel permeation chromatography (eluent: CHCl_3_) or preparative TLC (eluent: EtOAc/hexane). ^1^H and ^13^C{^1^H} NMR spectra of isolated products **5** are included in the Supplementary Materials.

Cyclohexyl(phenyl)selane (**5a**) (CAS: 22233-91-6) [44]. Pale-yellow liquid, 62.4 mg, 87%; ^1^H NMR (400 MHz, CDCl_3_): δ 7.56–7.53 (m, 2H, 2 × C*H*_arom_); 7.28–7.25 (m, 3H, 3 × C*H*_arom_), 3.29–3.22 (m, 1H, C*H*), 2.04–1.20 (m, 10H, 5 × C*H*_2_); ^13^C{^1^H} NMR (100 MHz, CDCl_3_): δ 134.7 (*C*H_arom_), 129.3 (*C*_arom_), 128.8 (*C*H_arom_), 127.2 (*C*H_arom_), 43.2 (*C*H), 34.2 (*C*H_2_), 26.9 (*C*H_2_), 25.7 (*C*H_2_); MS (EI) [M]^+^ *m*/*z* = 240.

(4-Chlorophenyl)(cyclohexyl)selane (**5b**). Pale-yellow liquid, 83.9 mg, 98%; ^1^H NMR (400 MHz, CDCl_3_): δ 7.48–7.44 (m, 2H, 2 × C*H*_arom_); 7.24–7.20 (m, 2H, 2 × C*H*_arom_), 3.25–3.18 (m, 1H, C*H*), 2.03–1.20 (m, 10H, 5 × C*H*_2_); ^13^C{^1^H} NMR (100 MHz, CDCl_3_): δ 136.1 (*C*H_arom_), 133.5 (*C*_arom_), 129.0 (*C*H_arom_), 127.4 (*C*_arom_), 43.6 (*C*H), 34.1 (*C*H_2_), 26.8 (*C*H_2_), 25.6 (*C*H_2_); ^77^Se{^1^H} NMR (76 MHz, CDCl_3_): δ 399 (br) [45]; IR (NaCl, ν/cm^−1^): 730, 815, 1011, 1090, 1447, 1457, 1472, 1506, 1558, 2850, 2928; HRMS (EI) *m*/*z* calcd for C_12_H_15_ClSe [M]^+^: 274.0025, found: 274.0023.

Cyclohexyl(4-fluorophenyl)selane (**5c**). Colorless liquid, 38.7 mg, 50%; ^1^H NMR (400 MHz, CDCl_3_): δ 7.55–7.50 (m, 2H, 2 × C*H*_arom_); 6.99–6.93 (m, 2H, 2 × C*H*_arom_), 3.20–3.13 (m, 1H, C*H*), 2.01–1.19 (m, 10H, 5 × C*H*_2_); ^13^C{^1^H} NMR (100 MHz, CDCl_3_): δ 162.6 (d, *J*_C-F_ = 247.3 Hz, *C*_arom_), 137.3 (d, *J*_C-F_ = 7.7 Hz, *C*H_arom_), 123.5 (d, *J*_C-F_ = 3.8 Hz, *C*_arom_), 116.0 (d, *J*_C-F_ = 22.0 Hz, *C*H_arom_), 43.7 (*C*H), 34.1 (*C*H_2_), 26.8 (*C*H_2_), 25.7 (*C*H_2_); ^19^F{^1^H} NMR (373 MHz, CDCl_3_): δ −114.5; IR (NaCl, ν/cm^−1^): 593, 811, 826, 1155, 1228, 1447, 1486, 1506, 1583, 2852, 2929.

Cyclohexyl(4-(trifluoromethyl)phenyl)selane (**5d**). Colorless liquid, 63.4 mg, 69%; ^1^H NMR (400 MHz, CDCl_3_): δ 7.61–7.59 (m, 2H, 2 × C*H*_arom_); 7.50–7.48 (m, 2H, 2 × C*H*_arom_), 3.41–3.34 (m, 1H, C*H*), 2.07–1.24 (m, 10H, 5 × C*H*_2_); ^13^C{^1^H} NMR (100 MHz, CDCl_3_): δ 135.1 (*C*_arom_), 133.5 (*C*H_arom_), 128.9 (q, *J*_C-F_ = 32.6 Hz, *C*_arom_), 125.5 (q, *J*_C-F_ = 2.9 Hz, *C*H_arom_), 124.2 (q, *J*_C-F_ = 271.6 Hz, *C*F_3_), 43.3 (*C*H), 34.1 (*C*H_2_), 26.8 (*C*H_2_), 25.7 (*C*H_2_); ^19^F{^1^H} NMR (373 MHz, CDCl_3_): δ −62.5; IR (NaCl, ν/cm^−1^): 687, 774, 821, 992, 1014, 1058, 1078, 1326, 1602, 2933; HRMS (EI) *m*/*z* calcd for C_12_H_15_ClSe [M]^+^: 274.0025, found: 274.0031.

(3-Chlorophenyl)(cyclohexyl)selane (**5e**). Colorless liquid, 46.8 mg, 57%; ^1^H NMR (400 MHz, CDCl_3_): δ 7.53 (t, *J* = 1.8 Hz, 1H, C*H*_arom_); 7.40 (dt, *J* = 7.3, 1.4 Hz, 1H, C*H*_arom_), 7.26–7.15 (m, 2H, 2 × C*H*_arom_), 3.32–3.25 (m, 1H, C*H*), 2.05–1.22 (m, 10H, 5 × C*H*_2_); ^13^C{^1^H} NMR (100 MHz, CDCl_3_): δ 134.3 (*C*_arom_), 133.9 (*C*H_arom_), 132.4 (*C*H_arom_), 131.1 (*C*_arom_), 129.8 (*C*H_arom_), 127.3 (*C*H_arom_), 43.6 (*C*H), 34.1 (*C*H_2_), 26.8 (*C*H_2_), 25.7 (*C*H_2_); IR (NaCl, ν/cm^−1^): 679, 754, 776, 992, 1447, 1458, 1570, 2851, 2928.

Cyclohexyl(3-fluorophenyl)selane (**5f**). Colorless liquid, 71.2 mg, 92%; ^1^H NMR (400 MHz, CDCl_3_): δ 7.30–7.17 (m, 3H, 3 × C*H*_arom_); 6.97–6.92 (m, 1H, C*H*_arom_), 3.33–3.26 (m, 1H, C*H*), 2.05–1.22 (m, 10H, 5 × C*H*_2_); ^13^C{^1^H} NMR (100 MHz, CDCl_3_): δ 162.4 (d, *J*_C-F_ = 249.2 Hz, *C*_arom_), 131.3 (d, *J*_C-F_ = 6.7 Hz, *C*_arom_), 130.0 (d, *J*_C-F_ = 8.6 Hz, *C*H_arom_), 129.7 (d, *J*_C-F_ = 2.9 Hz, *C*H_arom_), 120.8 (d, *J*_C-F_ = 21.1 Hz, *C*H_arom_), 114.1 (d, *J*_C-F_ = 21.1 Hz, *C*H_arom_), 43.5 (*C*H), 34.1 (*C*H_2_), 26.8 (*C*H_2_), 25.7 (*C*H_2_); ^19^F{^1^H} (373 MHz, CDCl_3_): δ −112.6; IR (NaCl, ν/cm^−1^): 777, 860, 1210, 1259, 1470, 1573, 2929.

(2-Chlorophenyl)(cyclohexyl)selane (**5h**). Pale-yellow liquid, 30.4 mg, 37%; ^1^H NMR (400 MHz, CDCl_3_): δ 7.49–7.45 (m, 1H, C*H*_arom_); 7.40–7.36 (m, 1H, C*H*_arom_), 7.19–7.14 (m, 2H, 2 × C*H*_arom_), 3.48–3.41 (m, 1H, C*H*), 2.08–1.25 (m, 10H, 5 × C*H*_2_); ^13^C{^1^H} NMR (100 MHz, CDCl_3_): δ 136.5 (*C*_arom_), 133.4 (*C*H_arom_), 130.8 (*C*_arom_), 129.6 (*C*H_arom_), 127.7 (*C*H_arom_), 127.0 (*C*H_arom_), 42.2 (*C*H), 33.8 (*C*H_2_), 26.8 (*C*H_2_), 25.8 (*C*H_2_); IR (NaCl, ν/cm^−1^): 744, 1024, 1447, 1634, 2851, 2929, 3390.

Cyclohexyl(2,3-dichlorophenyl)selane (**5i**). Yellow liquid, 43.4 mg, 47%; ^1^H NMR (400 MHz, CDCl_3_): δ 7.32 (dt, *J* = 1.4, 7.8 Hz, 2H, 2 × C*H*_arom_), 7.09 (t, *J* = 7.8 Hz, 1H, C*H*_arom_), 3.48–3.41 (m, 1H, C*H*), 2.08–1.25 (m, 10H, 5 × C*H*_2_); ^13^C{^1^H} NMR (100 MHz, CDCl_3_): δ 133.7 (*C*_arom_), 133.3 (*C*_arom_), 131.4 (*C*_arom_), 130.3 (*C*H_arom_), 128.1 (*C*H_arom_), 127.4 (*C*H_arom_), 42.6 (*C*H), 33.6 (*C*H_2_), 26.7 (*C*H_2_), 25.7 (*C*H_2_); IR (NaCl, ν/cm^−1^): 765, 1181, 1257, 1391, 1432, 1558, 2331, 2851, 2929.

(3-Chloro-4-fluorophenyl)(cyclohexyl)selane (**5j**). Colorless liquid, 62.2 mg, 71%; ^1^H NMR (400 MHz, CDCl_3_): δ 7.60 (dd, *J* = 2.0, 7.1 Hz, 1H, C*H*_arom_); 7.42–7.39 (m, 1H, C*H*_arom_), 7.03 (t, *J* = 8.9 Hz, 1H, C*H*_arom_), 3.34–3.17 (m, 1H, C*H*), 2.02–1.20 (m, 10H, 5 × C*H*_2_); ^13^C{^1^H} NMR (100 MHz, CDCl_3_): δ 157.8 (d, *J*_C-F_ = 249.2 Hz, *C*_arom_), 137.0 (*C*H_arom_), 135.1 (d, *J*_C-F_ = 6.7 Hz, *C*H_arom_), 124.6 (d, *J*_C-F_ = 3.8 Hz, *C*_arom_), 121.1 (d, *J*_C-F_ = 18.2 Hz, *C*_arom_), 116.9 (d, *J*_C-F_ = 21.1 Hz, *C*H_arom_), 44.1 (*C*H), 34.1 (*C*H_2_), 26.8 (*C*H_2_), 25.6 (*C*H_2_); ^19^F{^1^H} NMR (373 MHz, CDCl_3_): δ −116.7; IR (NaCl, ν/cm^−1^): 511, 706, 816, 1258, 1447, 1479, 2851, 2928.

Cyclohexyl(3,4-difluorophenyl)selane (**5k**). Colorless liquid, 74.5 mg, 90%; ^1^H NMR (400 MHz, CDCl_3_): δ 7.39–7.34 (m, 1H, C*H*_arom_); 7.28–7.24 (m, 1H, C*H*_arom_), 7.09–7.02 (m, 1H, C*H*_arom_), 3.34–3.17 (m, 1H, C*H*), 2.01–1.20 (m, 10H, 5 × C*H*_2_); ^13^C{^1^H} NMR (100 MHz, CDCl_3_): δ 151.3 (dd, *J*_C-F_ = 12.4, 20.0 Hz, *C*_arom_), 148.7 (dd, *J*_C-F_ = 12.9, 22.4 Hz, *C*_arom_), 131.4 (dd, *J*_C-F_ = 3.8, 5.7 Hz, *C*H_arom_), 124.3 (t, *J*_C-F_ = 4.8 Hz, *C*_arom_), 123.9 (d, *J*_C-F_ = 16.2 Hz, *C*H_arom_), 117.6 (d, *J*_C-F_ = 17.2 Hz, *C*H_arom_), 44.0 (*C*H), 34.1 (*C*H_2_), 26.8 (*C*H_2_), 25.6 (*C*H_2_). ^19^F{^1^H} NMR (373 MHz, CDCl_3_): δ −112.6; IR (NaCl, ν/cm^−1^): 626, 769, 812, 887, 1115, 1199, 1272, 1497, 1596, 2930, 3415.

## 4. Conclusions

In this paper, we report the novel organic reactions of diaryl dichalcogenides with hexacyclohexyldilead under photoirradiation to produce aryl cyclohexyl monochalcogenides. (PhSe)_2_ showed a high carbon-radical-capturing ability toward the cyclohexyl radicals generated from heavier-element compounds. Moreover, the aryl cyclohexyl selenides were formed in good yields. If the carbon-radical-capturing ability of (PhSe)_2_ was low, the disproportionation and dimerization of cyclohexyl radicals may have proceeded, resulting in the formation of cyclohexane, cyclohexene, and bicyclohexane. However, the high yields of aryl cyclohexyl selenides clearly indicate that (PhSe)_2_ acts as an excellent carbon-radical-trapping agent for alkyl radicals generated from heavier-element compounds.

In this reaction, hexacyclohexyldilead generates cyclohexyl radicals; however, to the best of our knowledge, radical initiators generating secondary alkyl radical are less common than those generating primary alkyl radicals (e.g., triethylborane and diethylzinc) and tertiary alkyl radicals (e.g., AIBN and V-40). In addition, compounds that behave as radical initiators at room temperature (e.g., V-70) typically need to be stored in a freezer. In contrast, hexacyclohexyldilead is stable at room temperature in the dark and efficiently generates cyclohexyl radicals upon photoirradiation. Therefore, hexacyclohexyldilead can be used as a novel and useful photochemical initiator. Further studies on radical chain reactions using hexacyclohexyldilead are currently in progress.

## Figures and Tables

**Table 1:**
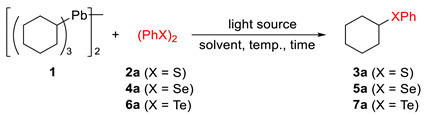
Reaction of diphenyl dichalcogenides with hexacyclohexyldilead **1** ^1^.

Entry	X	Light Source	Solvent	Temp., °C	Time, h	Yield, % ^2^
1	S	white LED	CHCl_3_	r.t.	24	26
2	S	xenon	CHCl_3_	r.t.	4	42
3	S	dark	toluene	100	12	trace
4 ^3^	S	dark	toluene	100	12	13
5	Se	white LED	CHCl_3_	r.t.	24	82
6	Se	blue LED	CHCl_3_	r.t.	24	80
7	Se	high-pressure Hg	CHCl_3_	r.t.	4	71
**8**	**Se**	**xenon**	**CHCl_3_**	**r.t.**	**4**	**93(87)**
9	Se	xenon	C_6_H_6_	r.t.	4	88
10	Se	xenon	MeCN	r.t.	4	69
11	Te	white LED	CHCl_3_	r.t.	24	37

^1^ Reaction conditions: [(C_6_H_11_)_3_Pb]_2_ (**1**; 0.05 mmol), (PhX)_2_ (**2a** (X = S), **4a** (X = Se), **6a** (X = Te); 0.3 mmol) and solvent (4 mL). ^2^ Determined by ^1^H NMR spectroscopy (isolated yield indicated in parentheses). ^3^ Benzoyl peroxide (0.5 equiv.) was added.

## Data Availability

The data presented in this study are available in the article and in its Supplementary Materials.

## Supplementary Materials

The following are available online, Copies of ^1^H and ^13^C{^1^H} NMR spectra.

## Sample Availability

Samples of the compounds are not available from the authors.
