# Supplementary material for: Clarification on the Reactivity of Diaryl Diselenides toward Hexacyclohexyldilead under Light
Source: Molecules. 2021 Oct 16;26(20):6265. doi: 10.3390/molecules26206265 (PMC8541589; doi:10.3390/molecules26206265)
Supplement: Supplementary file 1 [file molecules-26-06265-s001.zip › molecules-1399447-supplementary_corrected.pdf]

molecules

MDPI

Supplementary Materials

Clarification on the Reactivity of Diaryl Diselenides toward Hexacyclohexyldilead under Light

Vu Thai Hung, Cong Chi Tran, Yuki Yamamoto, Shintaro Kodama *, Akihiro Nomoto, and Akiya Ogawa *

Department of Applied Chemistry, Graduate School of Engineering, Osaka Prefecture University, 1-1 Gakuen-cho, Nakaku, Sakai, Osaka 599-8531, Japan; vth261@gmail.com (V.T.H.); mz105131@edu.osakafu-u.ac.jp (C.C.T.); syb02137@edu.osakafu-u.ac.jp (Y.Y.); nomoto@chem.osakafu-u.ac.jp (A.N.)

* Correspondence: skodama@chem.osakafu-u.ac.jp (S.K.); ogawa@chem.osakafu-u.ac.jp (A.O.)

Contents

Copies of ^{1}H and $^{13}C\{^{1}H\}$ NMR spectra ··· 2–12

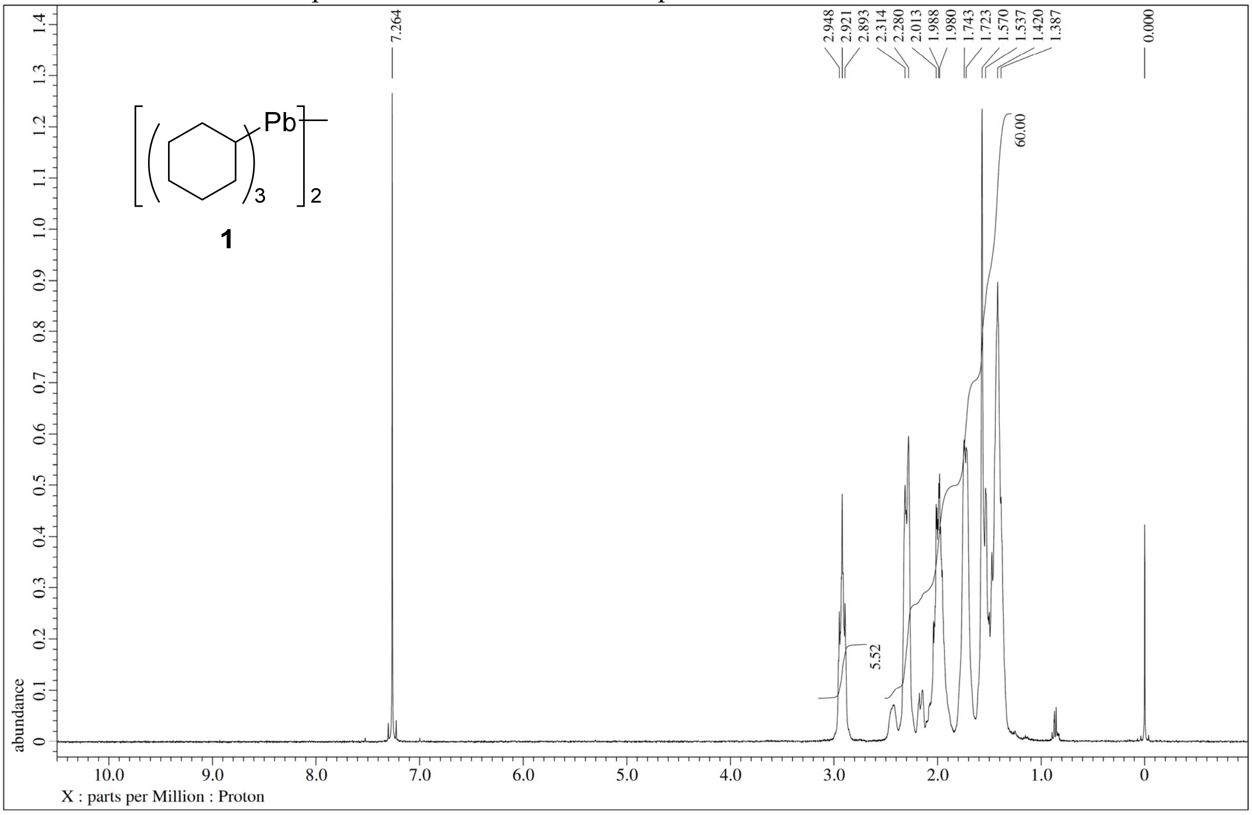

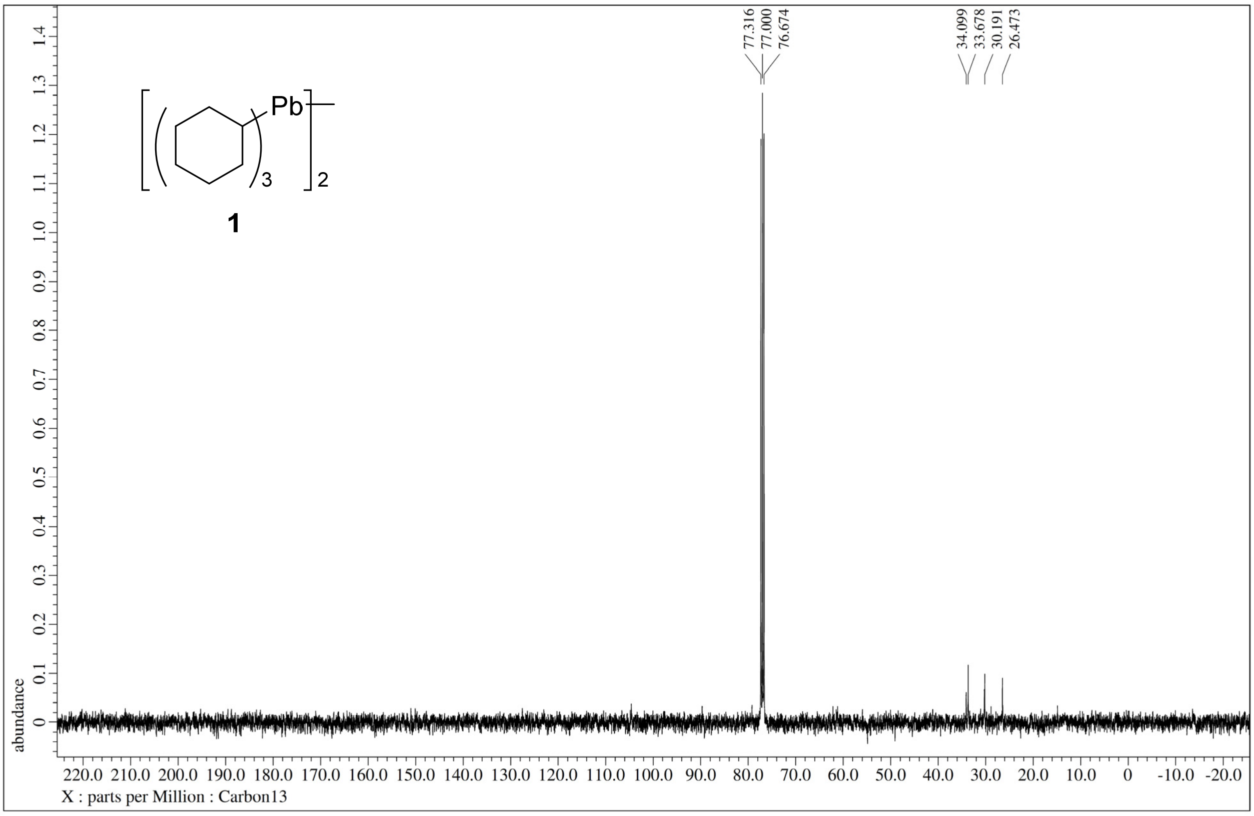
77.316
77.000
76.674
34.099
33.678
30.191
26.473
Pb
3
2
1
abundance
X : parts per Million : Carbon13

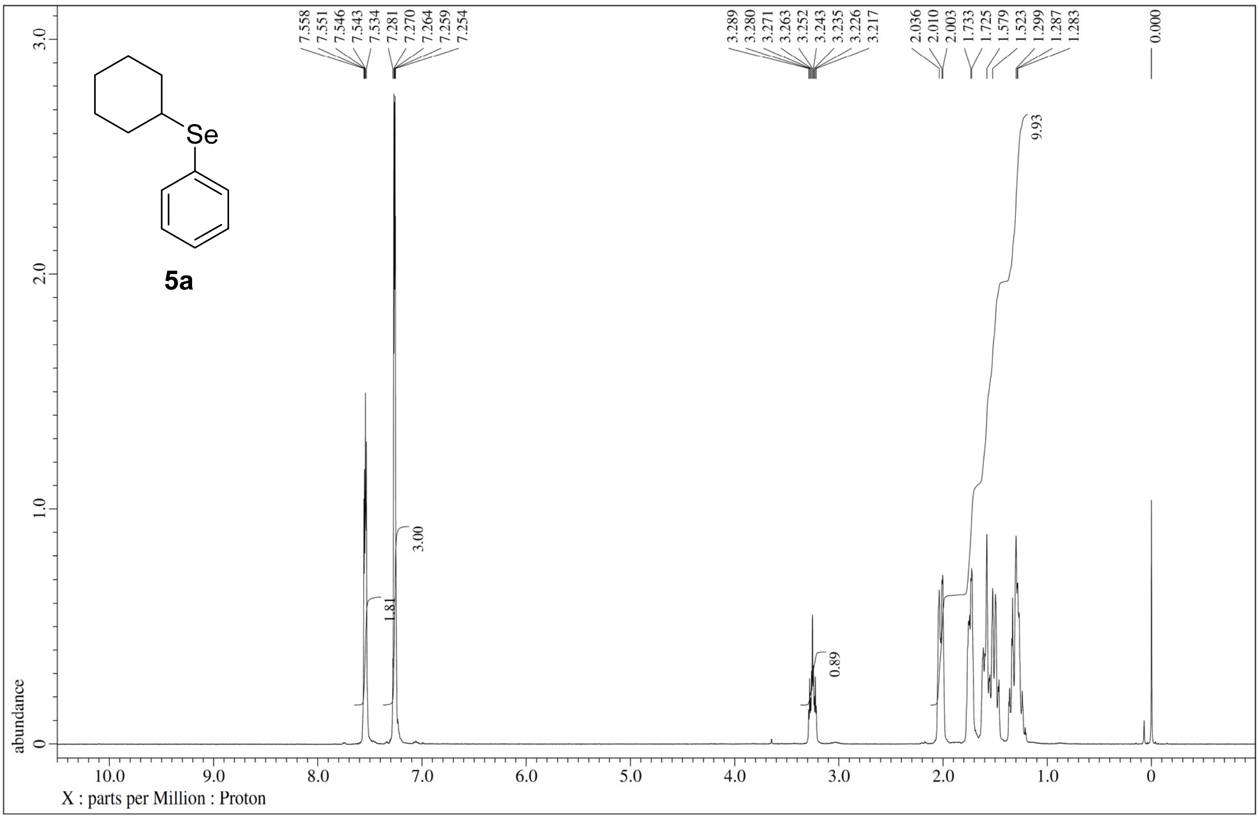
7.558
7.551
7.546
7.543
7.534
7.281
7.270
7.264
7.259
7.254
3.289
3.280
3.271
3.263
3.252
3.243
3.235
3.226
3.217
2.036
2.010
2.003
1.733
1.725
1.579
1.523
1.299
1.287
1.283
0.000
Se
5a
9.93
3.00
1.81
0.89
abundance
X : parts per Million : Proton

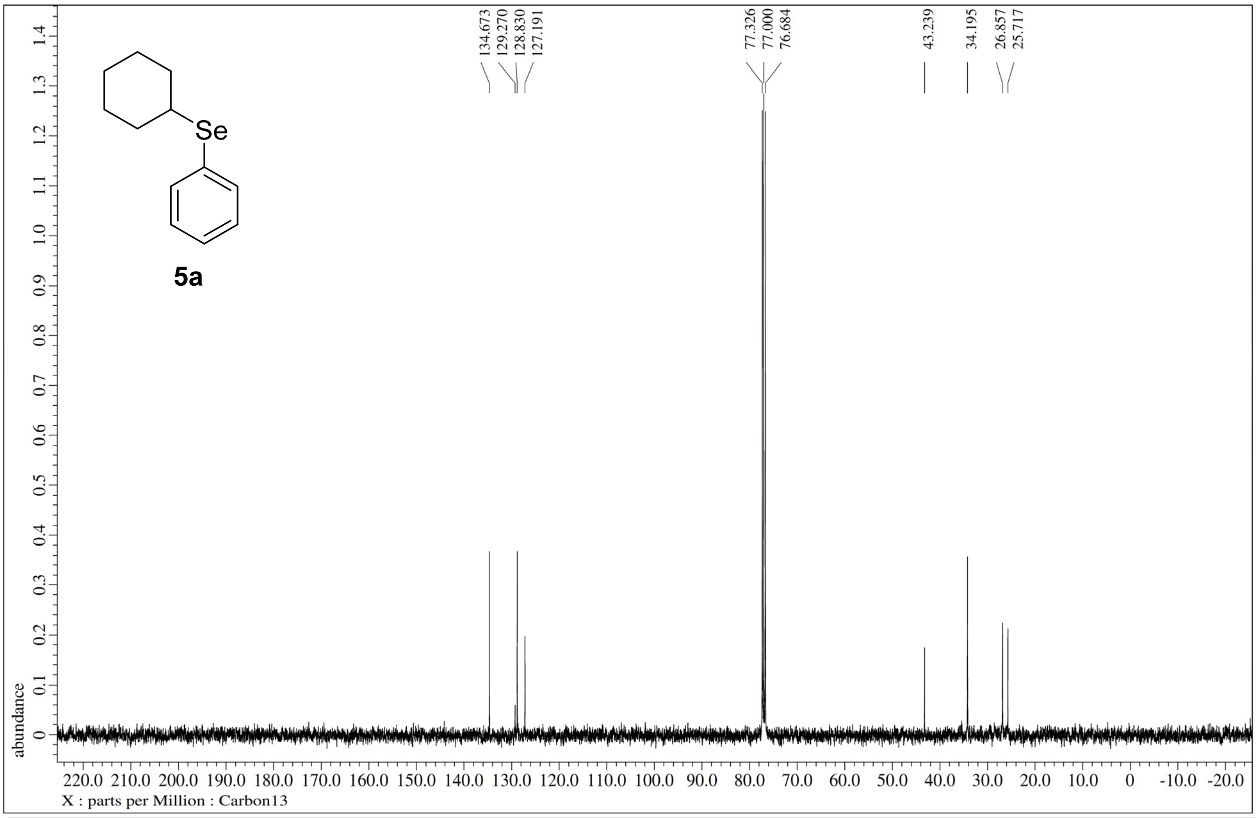
134.673
129.270
128.830
127.191
77.326
77.000
76.684
43.239
34.195
26.857
25.717
Se
5a
abundance
220.0 210.0 200.0 190.0 180.0 170.0 160.0 150.0 140.0 130.0 120.0 110.0 100.0 90.0 80.0 70.0 60.0 50.0 40.0 30.0 20.0 10.0 0 -10.0 -20.0
X : parts per Million : Carbon13

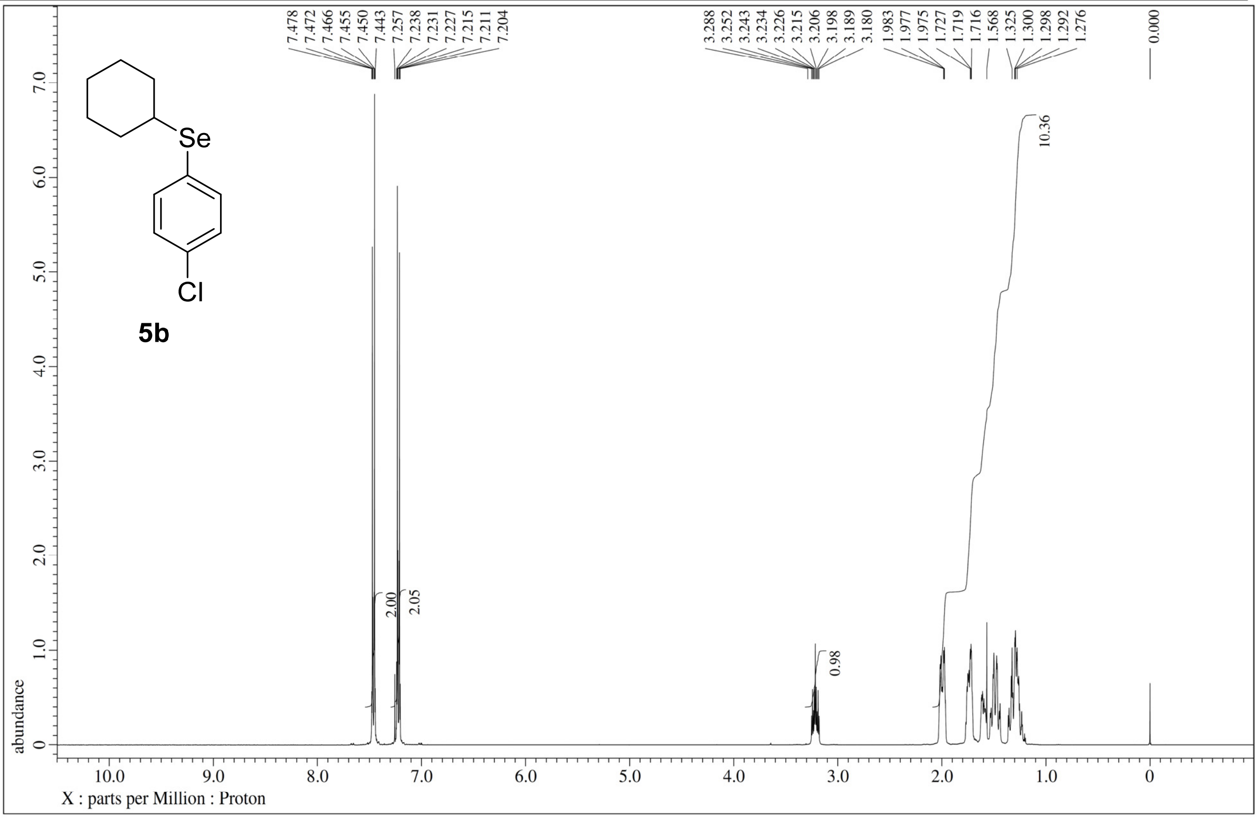
7.478
7.472
7.466
7.455
7.450
7.443
7.257
7.238
7.231
7.227
7.215
7.211
7.204
3.288
3.252
3.243
3.234
3.226
3.215
3.206
3.198
3.189
3.180
1.983
1.977
1.975
1.727
1.719
1.716
1.568
1.325
1.300
1.298
1.292
1.276
0.000
Se
Cl
5b
2.00
2.05
0.98
10.36
abundance
10.0 9.0 8.0 7.0 6.0 5.0 4.0 3.0 2.0 1.0 0
X : parts per Million : Proton

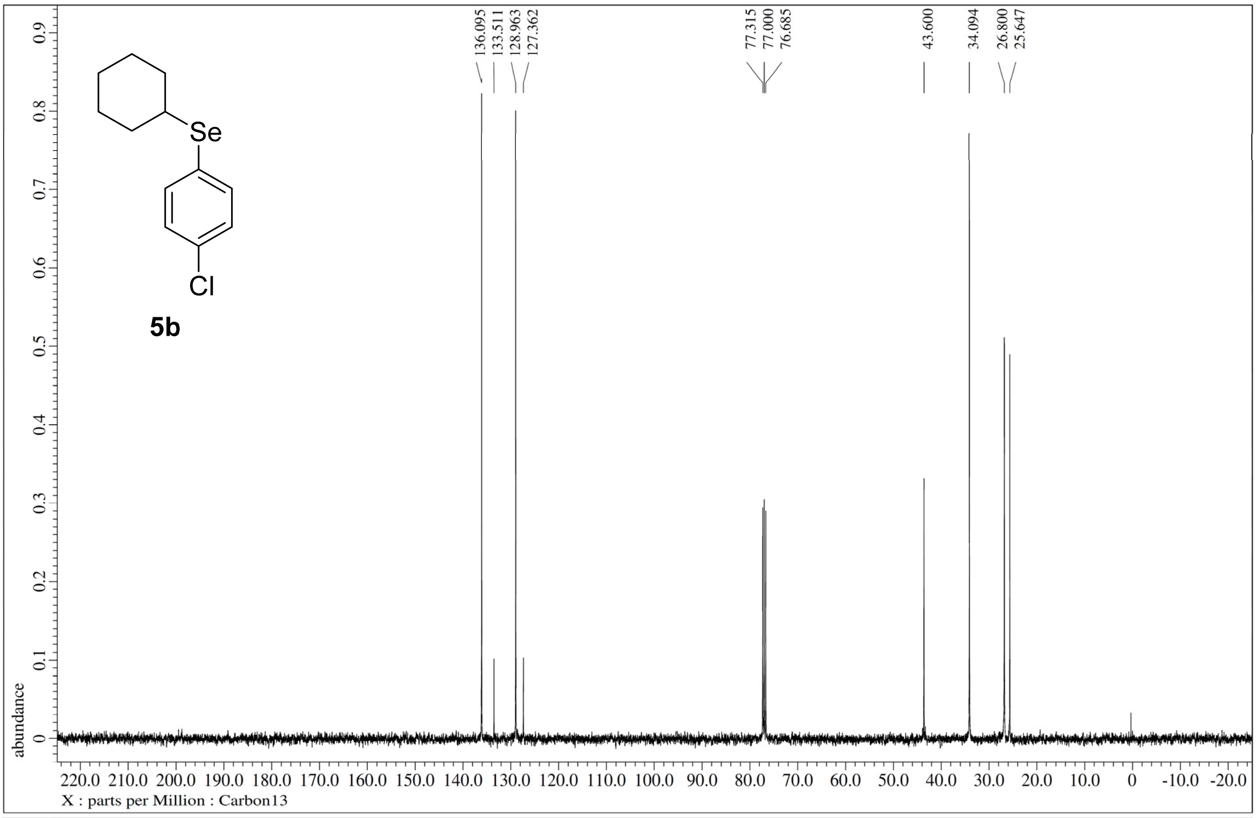
136.095
133.511
128.963
127.362
77.315
77.000
76.685
43.600
34.094
26.800
25.647
Se
Cl
5b
abundance
0 0.1 0.2 0.3 0.4 0.5 0.6 0.7 0.8 0.9
220.0 210.0 200.0 190.0 180.0 170.0 160.0 150.0 140.0 130.0 120.0 110.0 100.0 90.0 80.0 70.0 60.0 50.0 40.0 30.0 20.0 10.0 0 -10.0 -20.0
X : parts per Million : Carbon13

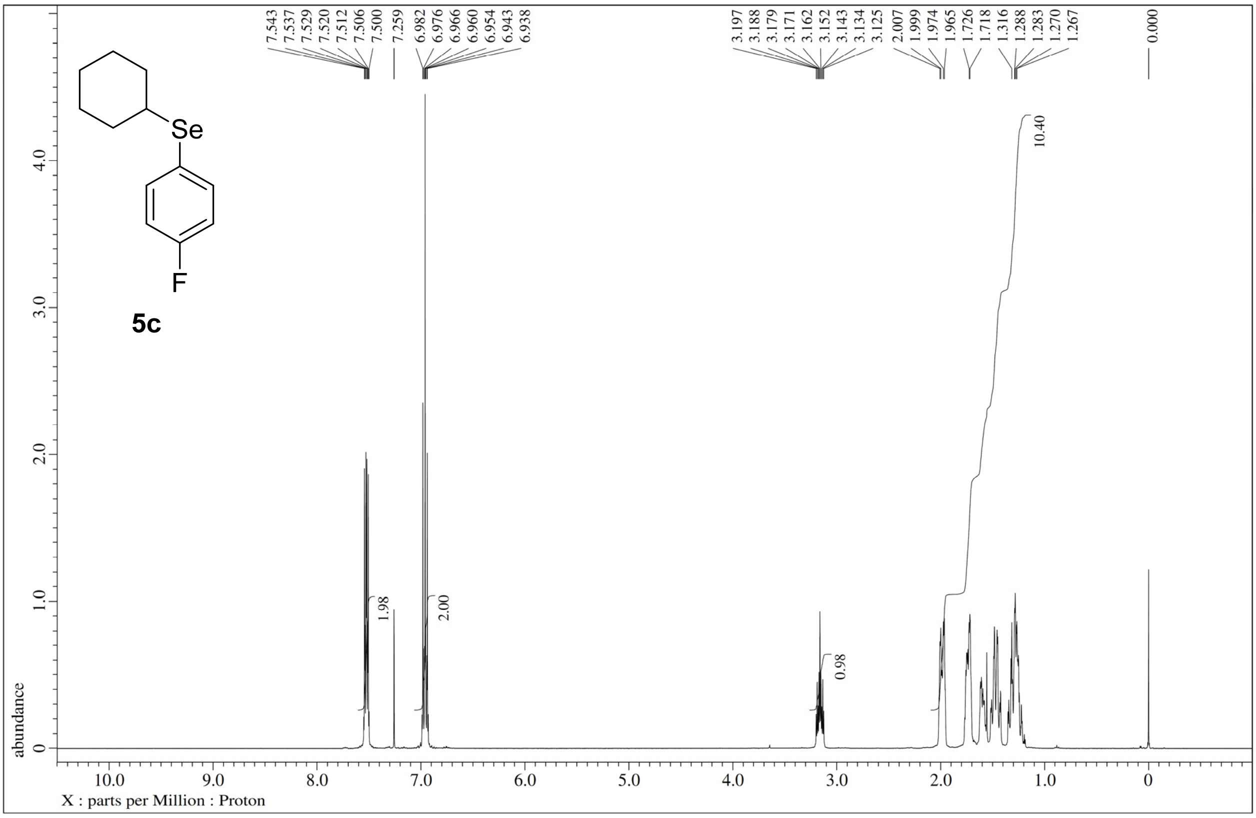
7.543
7.537
7.529
7.520
7.512
7.506
7.500
7.259
6.982
6.976
6.966
6.960
6.954
6.943
6.938
3.197
3.188
3.179
3.171
3.162
3.152
3.143
3.134
3.125
2.007
1.999
1.974
1.965
1.726
1.718
1.316
1.288
1.283
1.270
1.267
0.000
Se
F
5c
1.98
2.00
0.98
10.40
abundance
0 1.0 2.0 3.0 4.0
10.0 9.0 8.0 7.0 6.0 5.0 4.0 3.0 2.0 1.0 0
X : parts per Million : Proton

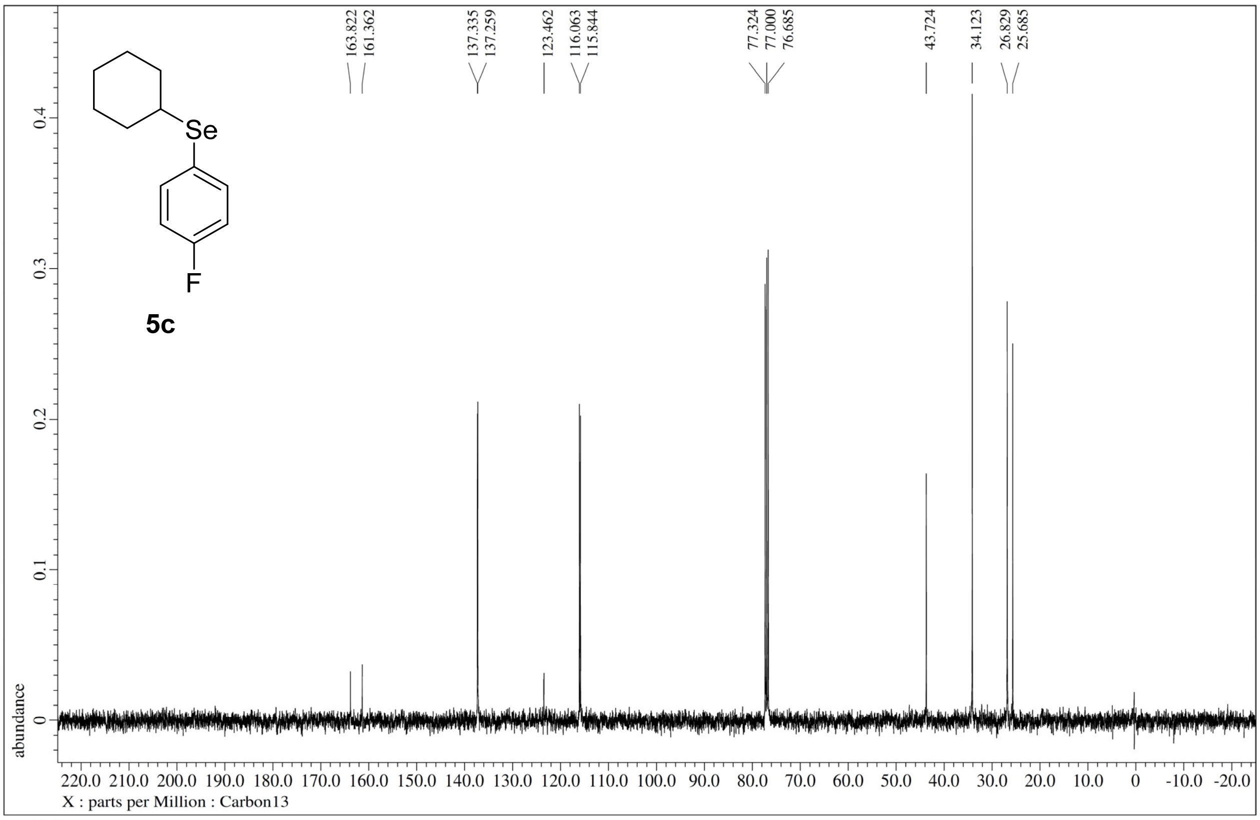
Se
F
5c
163.822
161.362
137.335
137.259
123.462
116.063
115.844
77.324
77.000
76.685
43.724
34.123
26.829
25.685
abundance
0
0.1
0.2
0.3
0.4
220.0 210.0 200.0 190.0 180.0 170.0 160.0 150.0 140.0 130.0 120.0 110.0 100.0 90.0 80.0 70.0 60.0 50.0 40.0 30.0 20.0 10.0 0 -10.0 -20.0
X : parts per Million : Carbon13

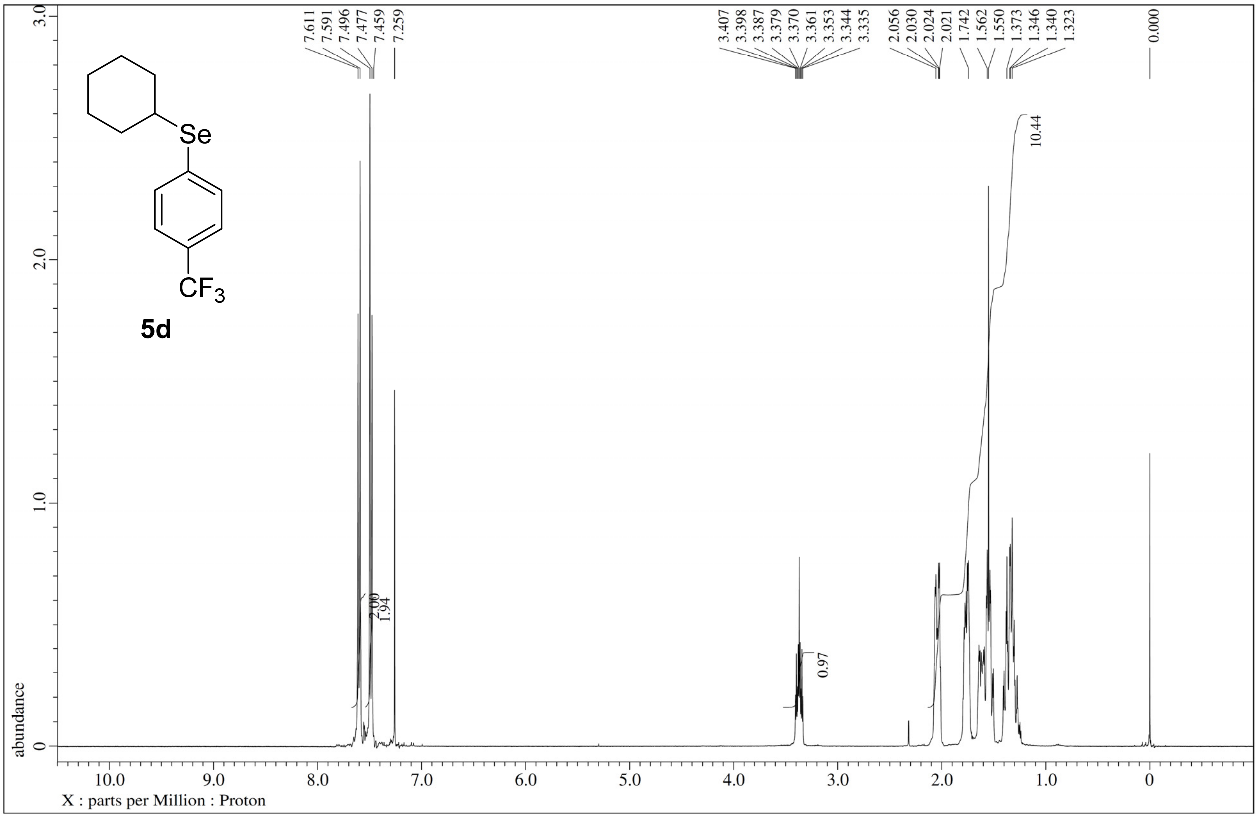
Se
CF3
5d
7.611
7.591
7.496
7.477
7.459
7.259
3.407
3.398
3.387
3.379
3.370
3.361
3.353
3.344
3.335
2.056
2.030
2.024
2.021
1.742
1.562
1.550
1.373
1.346
1.340
1.323
0.000
10.44
2.00
1.94
0.97
abundance
0
1.0
2.0
3.0
10.0 9.0 8.0 7.0 6.0 5.0 4.0 3.0 2.0 1.0 0
X : parts per Million : Proton

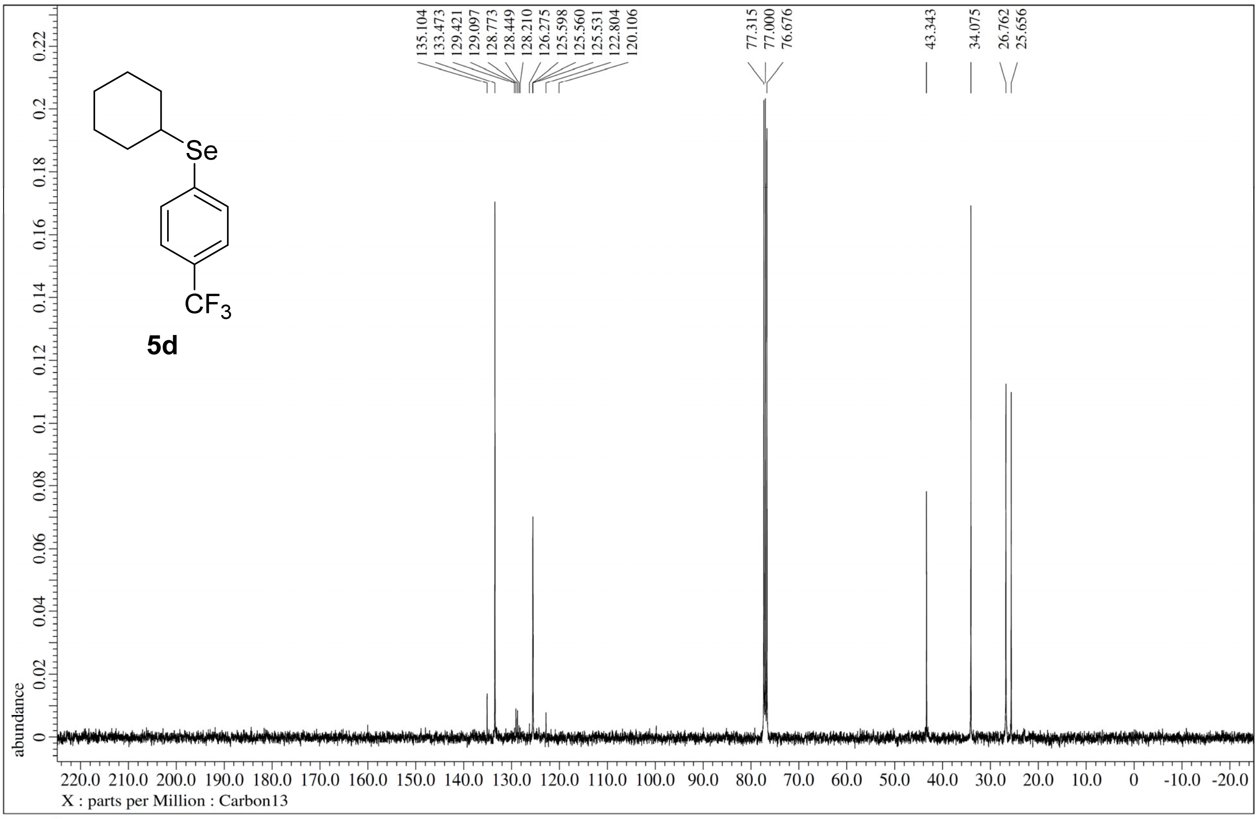
Se
CF3
5d
135.104
133.473
129.421
129.097
128.773
128.449
128.210
126.275
125.598
125.560
125.531
122.804
120.106
77.315
77.000
76.676
43.343
34.075
26.762
25.656
abundance
0
0.02
0.04
0.06
0.08
0.1
0.12
0.14
0.16
0.18
0.2
0.22
220.0 210.0 200.0 190.0 180.0 170.0 160.0 150.0 140.0 130.0 120.0 110.0 100.0 90.0 80.0 70.0 60.0 50.0 40.0 30.0 20.0 10.0 0 -10.0 -20.0
X : parts per Million : Carbon13

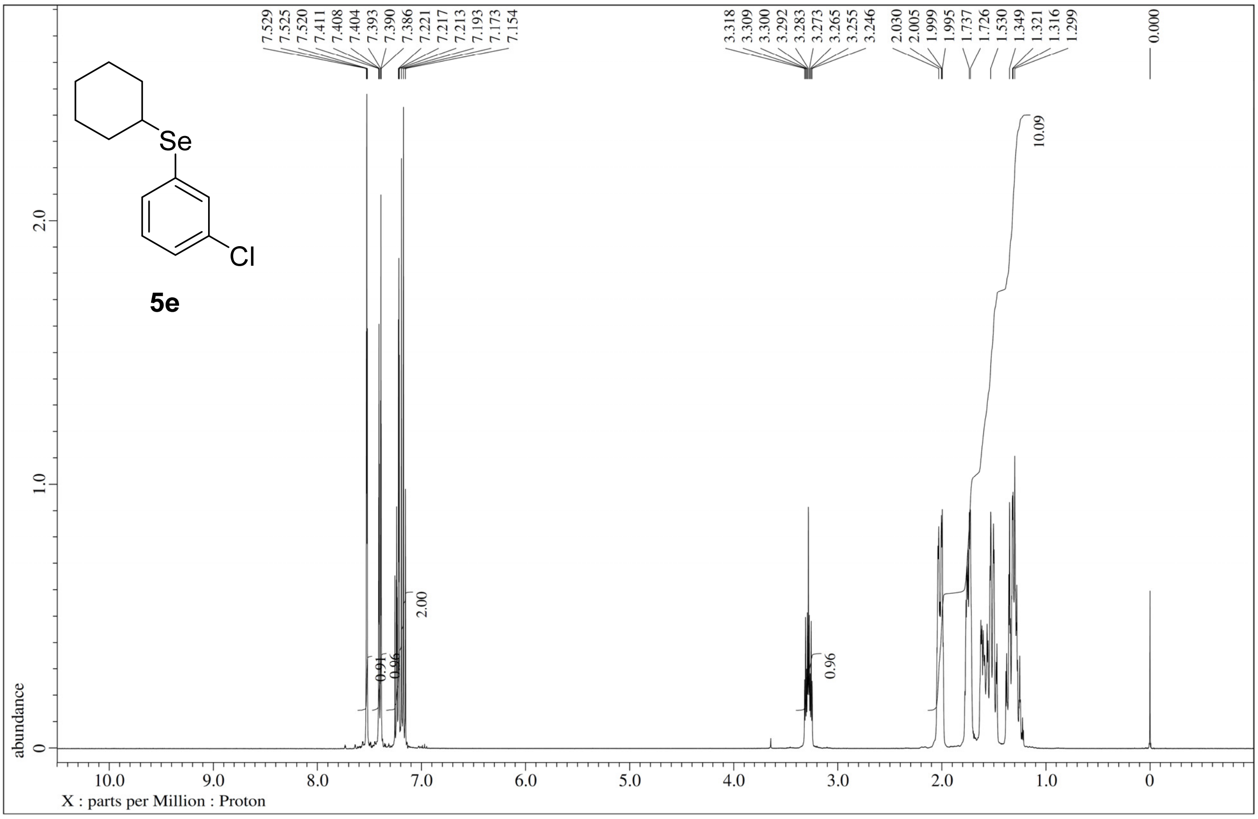
Se
Cl
5e
7.529
7.525
7.520
7.411
7.408
7.404
7.393
7.390
7.386
7.221
7.217
7.213
7.193
7.173
7.154
3.318
3.309
3.300
3.292
3.283
3.273
3.265
3.255
3.246
2.030
2.005
1.999
1.995
1.737
1.726
1.530
1.349
1.321
1.316
1.299
0.000
10.09
2.00
0.91
0.96
0.96
abundance
0
1.0
2.0
10.0 9.0 8.0 7.0 6.0 5.0 4.0 3.0 2.0 1.0 0
X : parts per Million : Proton

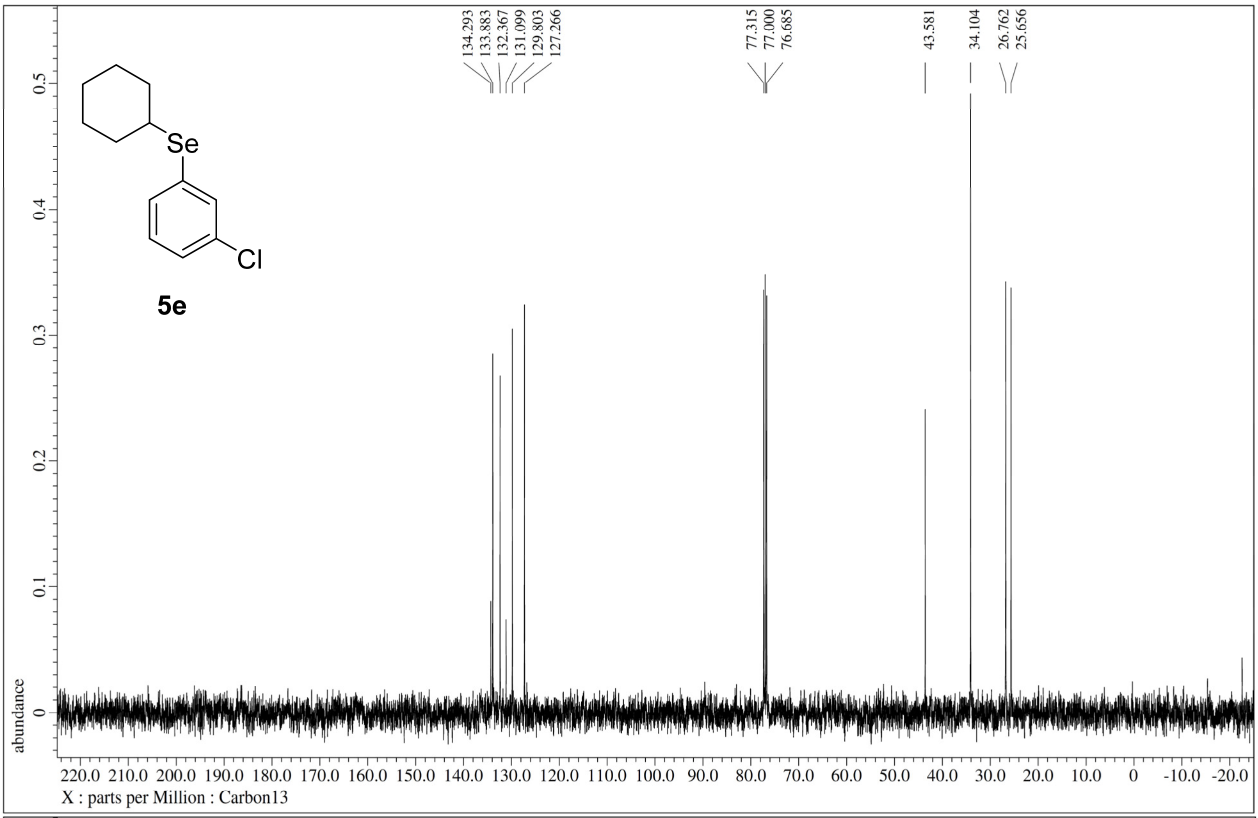

134.293
133.883
132.367
131.099
129.803
127.266
77.315
77.000
76.685
43.581
34.104
26.762
25.656
Se
Cl
5e
abundance
0 0.1 0.2 0.3 0.4 0.5
220.0 210.0 200.0 190.0 180.0 170.0 160.0 150.0 140.0 130.0 120.0 110.0 100.0 90.0 80.0 70.0 60.0 50.0 40.0 30.0 20.0 10.0 0 -10.0 -20.0
X : parts per Million : Carbon13

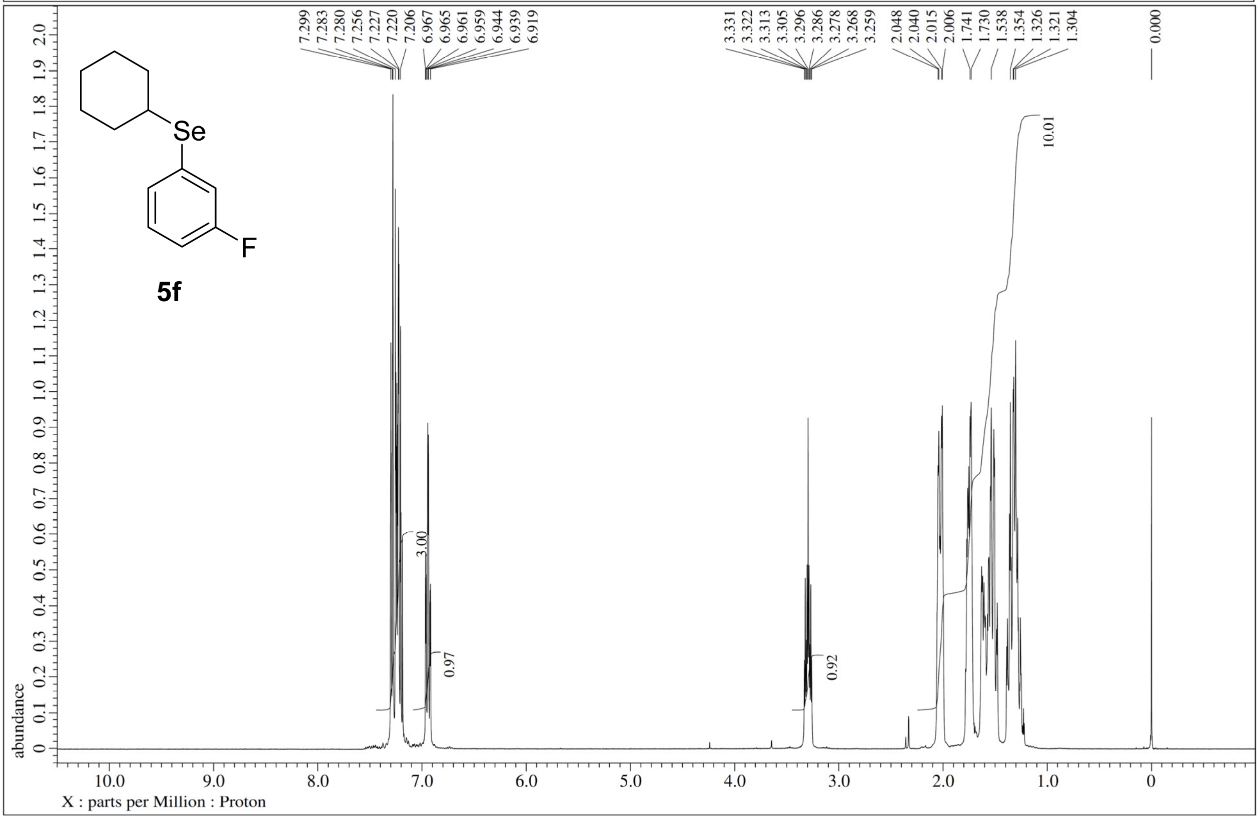

7.299
7.283
7.280
7.256
7.227
7.220
7.206
6.967
6.965
6.961
6.959
6.944
6.939
6.919
3.331
3.322
3.313
3.305
3.296
3.286
3.278
3.268
3.259
2.048
2.040
2.015
2.006
1.741
1.730
1.538
1.354
1.326
1.321
1.304
0.000
Se
F
5f
3.00
0.97
0.92
10.01
abundance
0 0.1 0.2 0.3 0.4 0.5 0.6 0.7 0.8 0.9 1.0 1.1 1.2 1.3 1.4 1.5 1.6 1.7 1.8 1.9 2.0
10.0 9.0 8.0 7.0 6.0 5.0 4.0 3.0 2.0 1.0 0
X : parts per Million : Proton

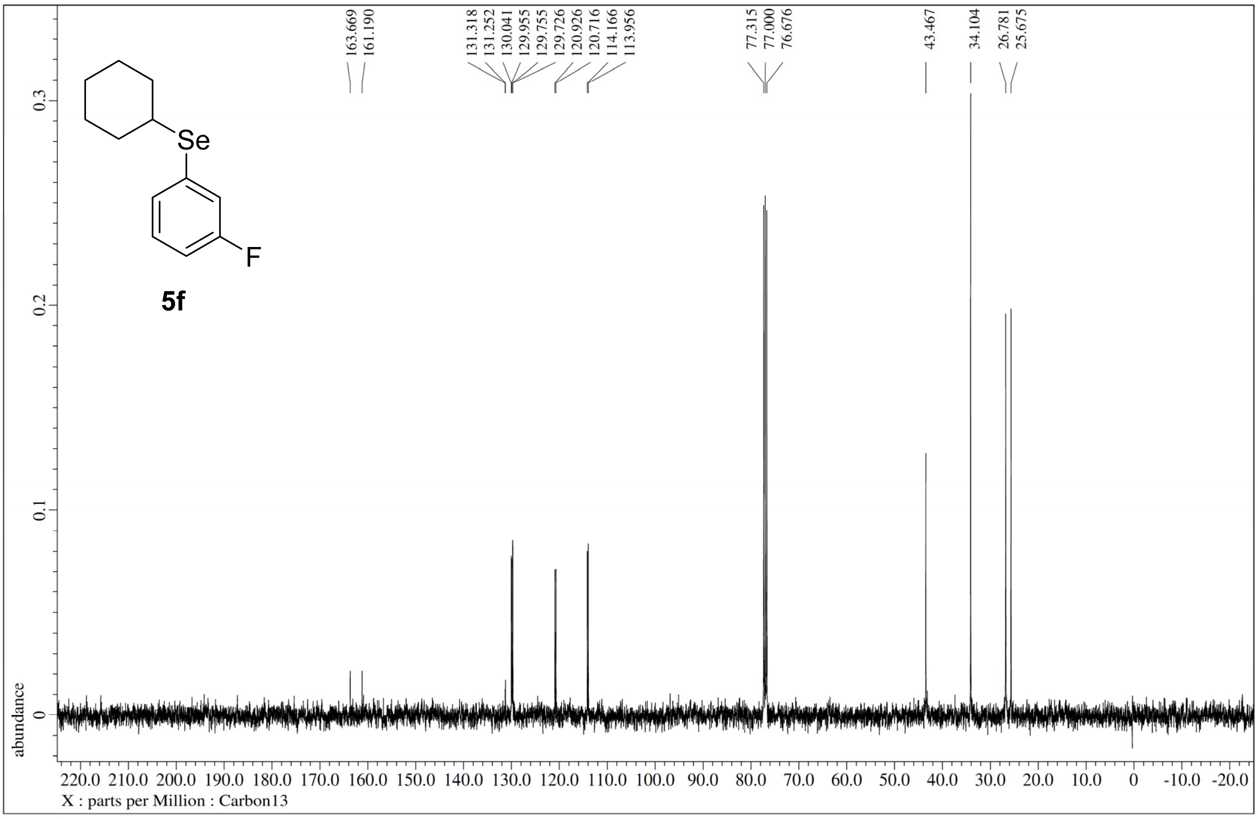

Se
F
5f
163.669
161.190
131.318
131.252
130.041
129.955
129.755
129.726
120.926
120.716
114.166
113.956
77.315
77.000
76.676
43.467
34.104
26.781
25.675
abundance
0.3
0.2
0.1
0
220.0 210.0 200.0 190.0 180.0 170.0 160.0 150.0 140.0 130.0 120.0 110.0 100.0 90.0 80.0 70.0 60.0 50.0 40.0 30.0 20.0 10.0 0 -10.0 -20.0
X : parts per Million : Carbon13

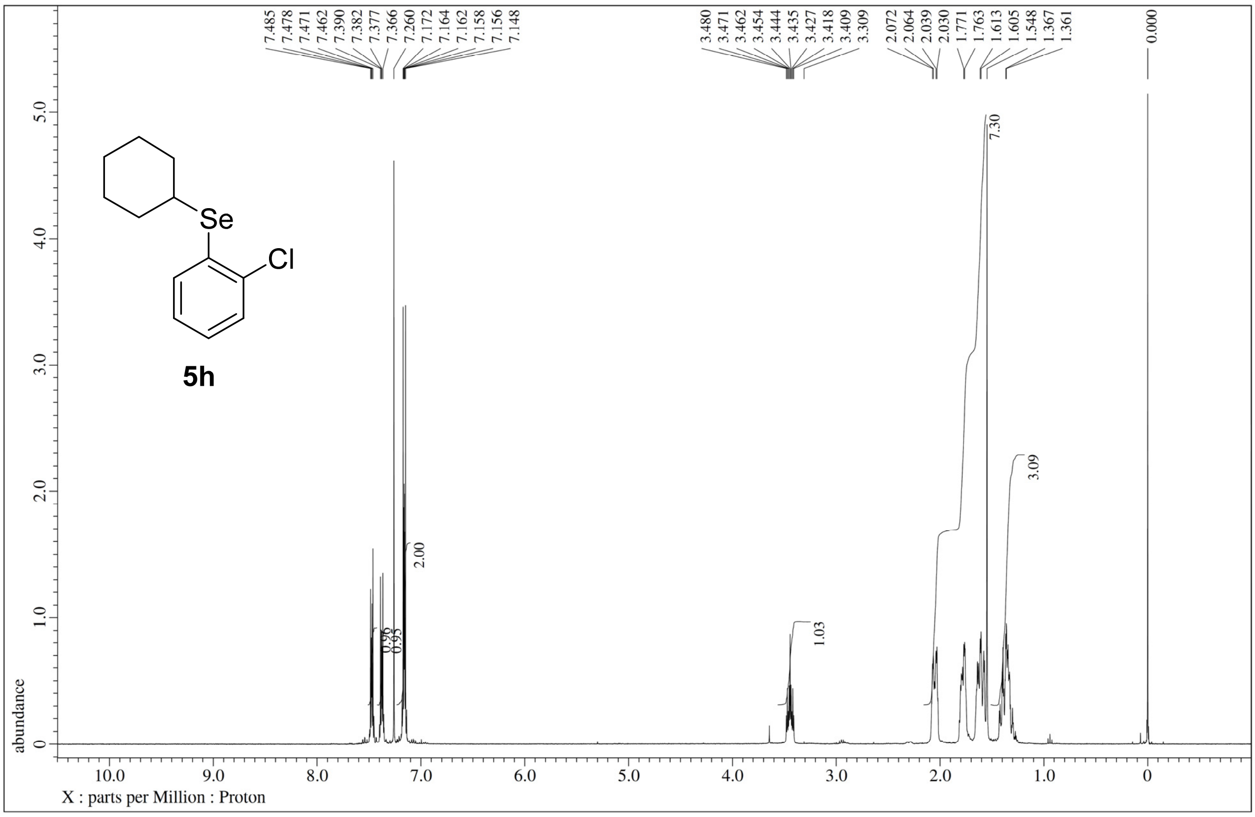

Se
Cl
5h
7.485
7.478
7.471
7.462
7.390
7.382
7.377
7.366
7.260
7.172
7.164
7.162
7.158
7.156
7.148
3.480
3.471
3.462
3.454
3.444
3.435
3.427
3.418
3.409
3.309
2.072
2.064
2.039
2.030
1.771
1.763
1.613
1.605
1.548
1.367
1.361
0.000
7.30
3.09
2.00
1.03
abundance
5.0
4.0
3.0
2.0
1.0
0
10.0 9.0 8.0 7.0 6.0 5.0 4.0 3.0 2.0 1.0 0
X : parts per Million : Proton

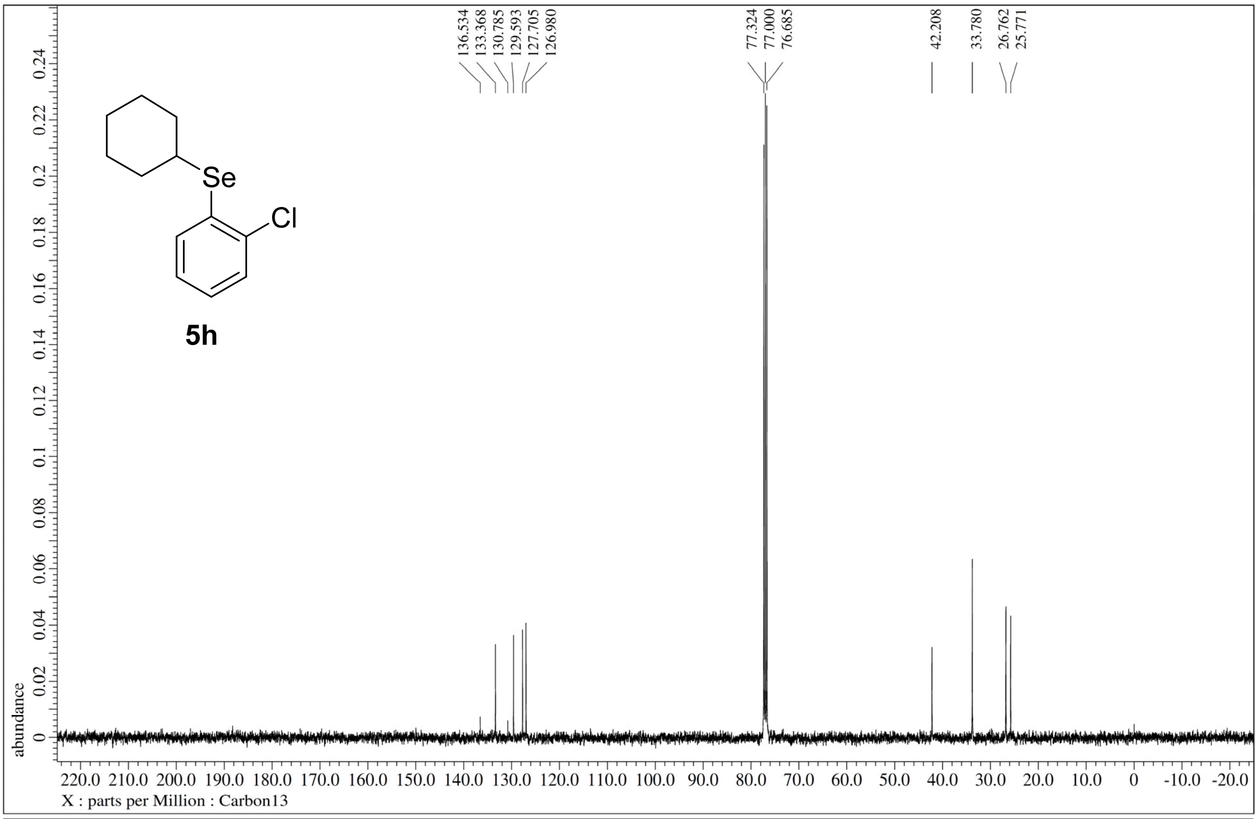
136.534
133.368
130.785
129.593
127.705
126.980
77.324
77.000
76.685
42.208
33.780
26.762
25.771
Se
Cl
5h
abundance
X : parts per Million : Carbon13

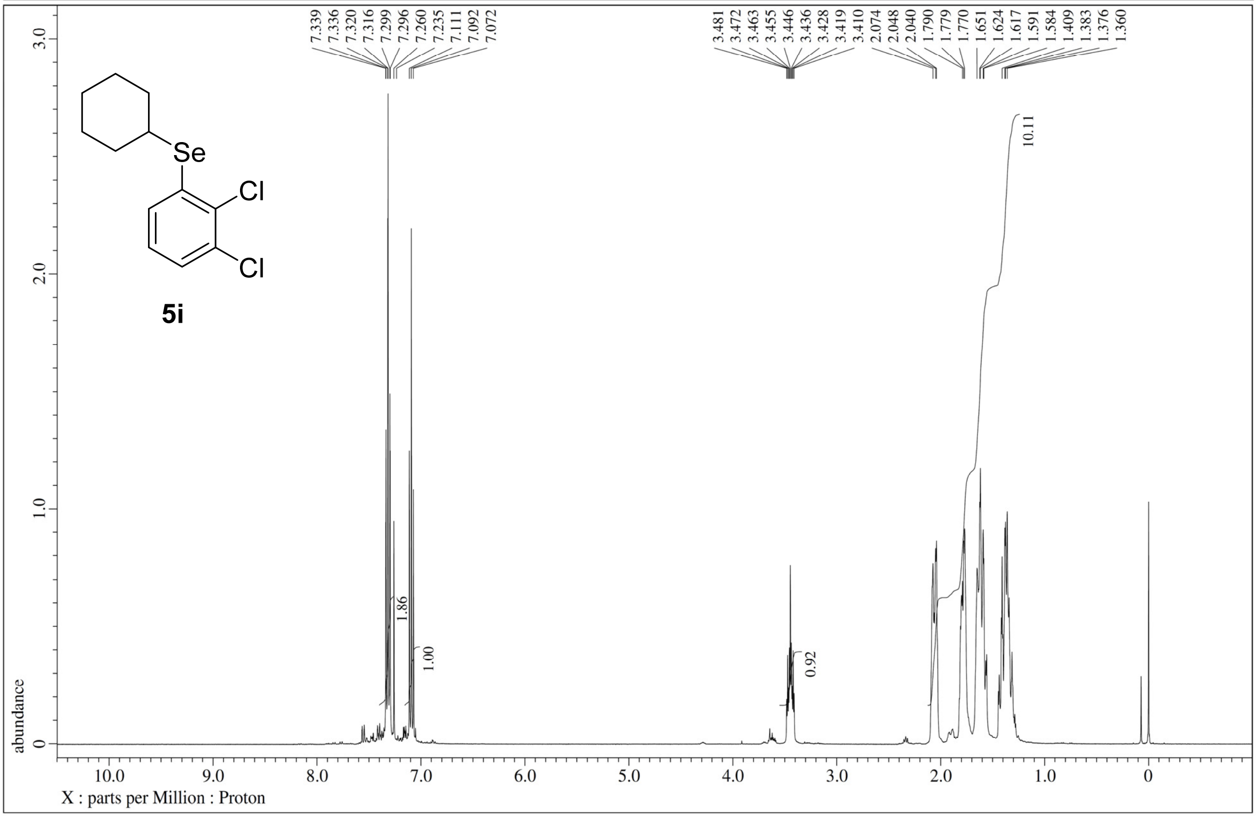
7.339
7.336
7.320
7.316
7.299
7.296
7.260
7.235
7.111
7.092
7.072
3.481
3.472
3.463
3.455
3.446
3.436
3.428
3.419
3.410
2.074
2.048
2.040
1.790
1.779
1.770
1.651
1.624
1.617
1.591
1.584
1.409
1.383
1.376
1.360
10.11
1.86
1.00
0.92
Se
Cl
Cl
5i
abundance
X : parts per Million : Proton

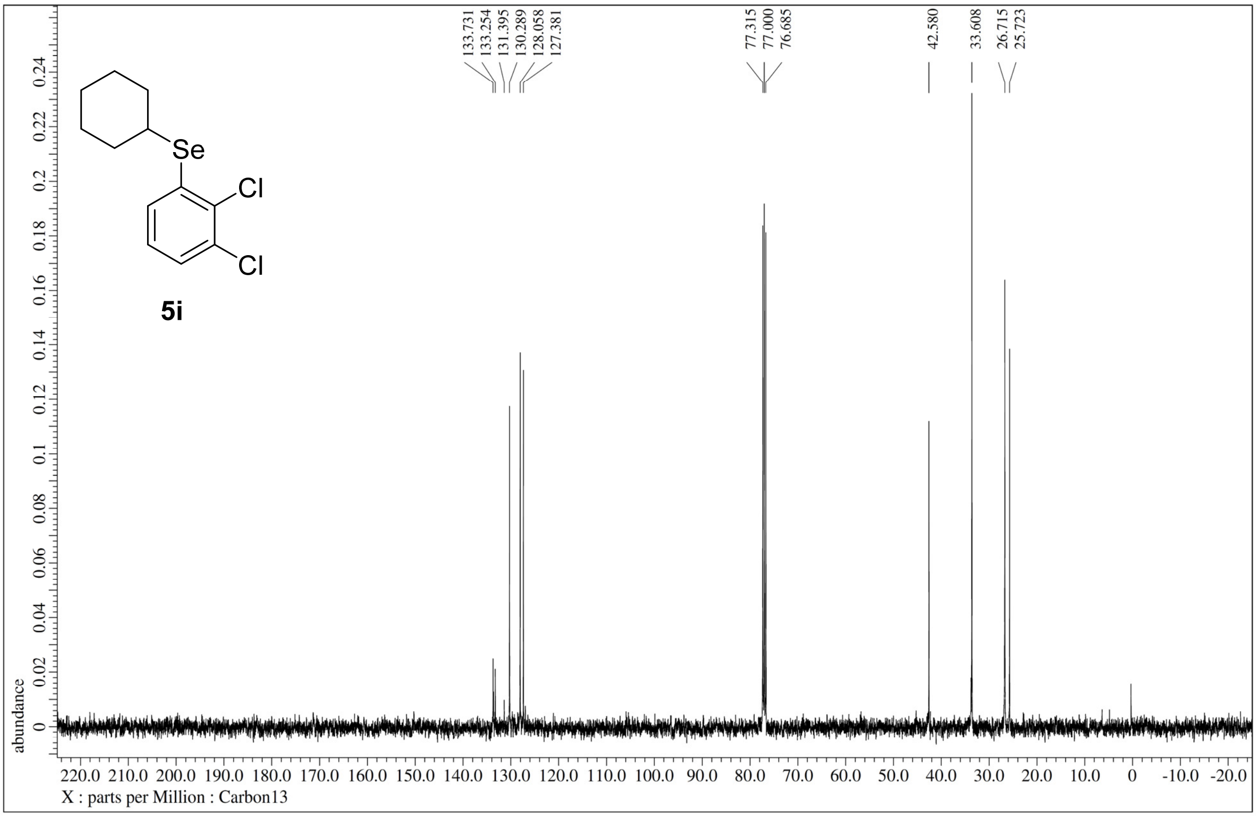
133.731
133.254
131.395
130.289
128.058
127.381
77.315
77.000
76.685
42.580
33.608
26.715
25.723
Se
Cl
Cl
5i
abundance
0 0.02 0.04 0.06 0.08 0.1 0.12 0.14 0.16 0.18 0.2 0.22 0.24
220.0 210.0 200.0 190.0 180.0 170.0 160.0 150.0 140.0 130.0 120.0 110.0 100.0 90.0 80.0 70.0 60.0 50.0 40.0 30.0 20.0 10.0 0 -10.0 -20.0
X : parts per Million : Carbon13

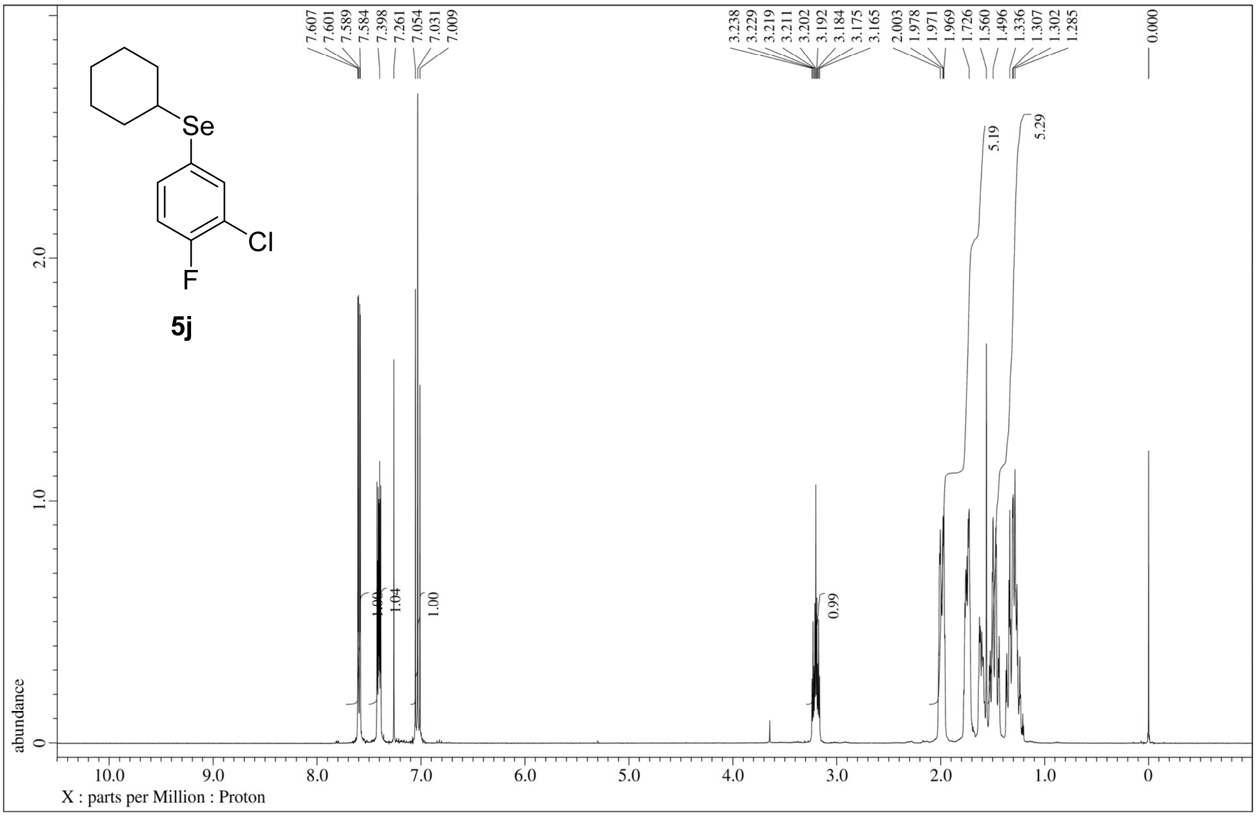
7.607
7.601
7.589
7.584
7.398
7.261
7.054
7.031
7.009
3.238
3.229
3.219
3.211
3.202
3.192
3.184
3.175
3.165
2.003
1.978
1.971
1.969
1.726
1.560
1.496
1.336
1.307
1.302
1.285
0.000
Se
Cl
F
5j
1.04
1.00
0.99
5.19
5.29
abundance
0 1.0 2.0
10.0 9.0 8.0 7.0 6.0 5.0 4.0 3.0 2.0 1.0 0
X : parts per Million : Proton

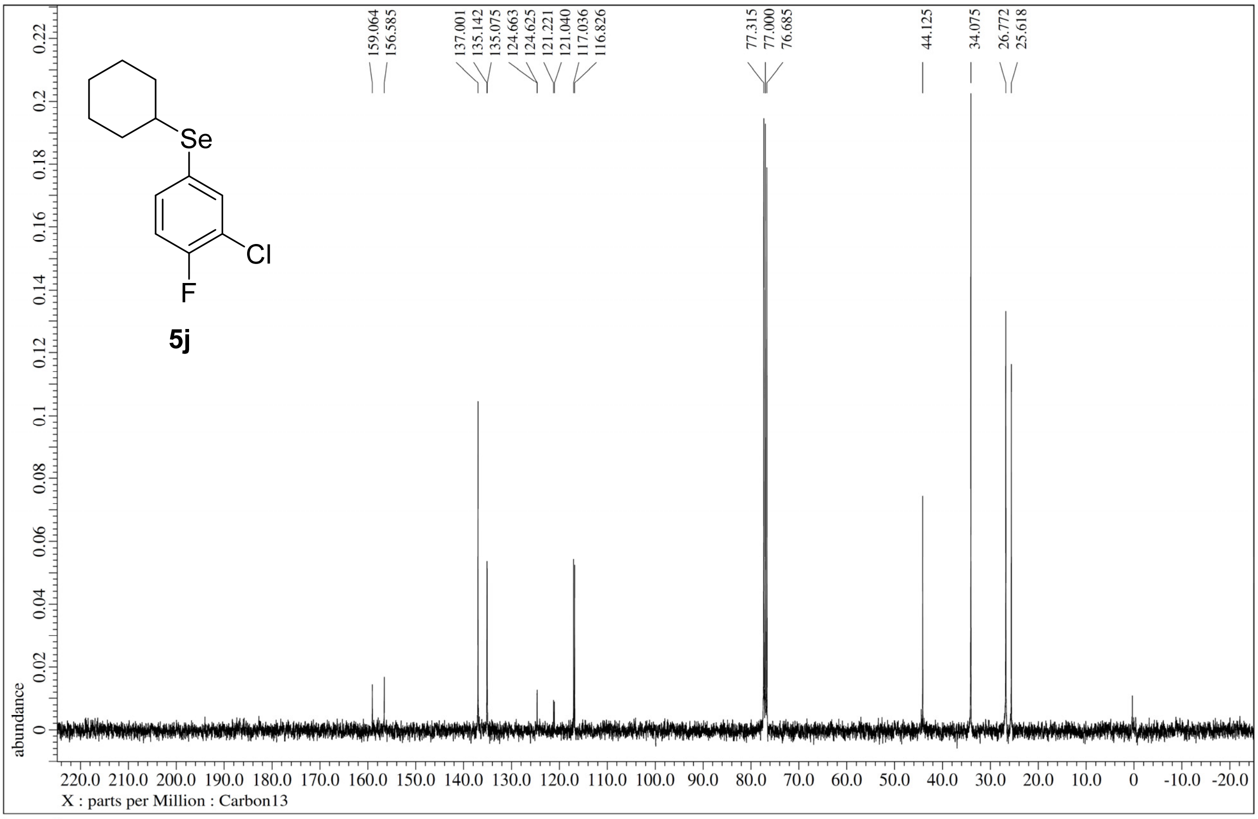
159.064
156.585
137.001
135.142
135.075
124.663
124.625
121.221
121.040
117.036
116.826
77.315
77.000
76.685
44.125
34.075
26.772
25.618
Se
Cl
F
5j
abundance
X : parts per Million : Carbon13

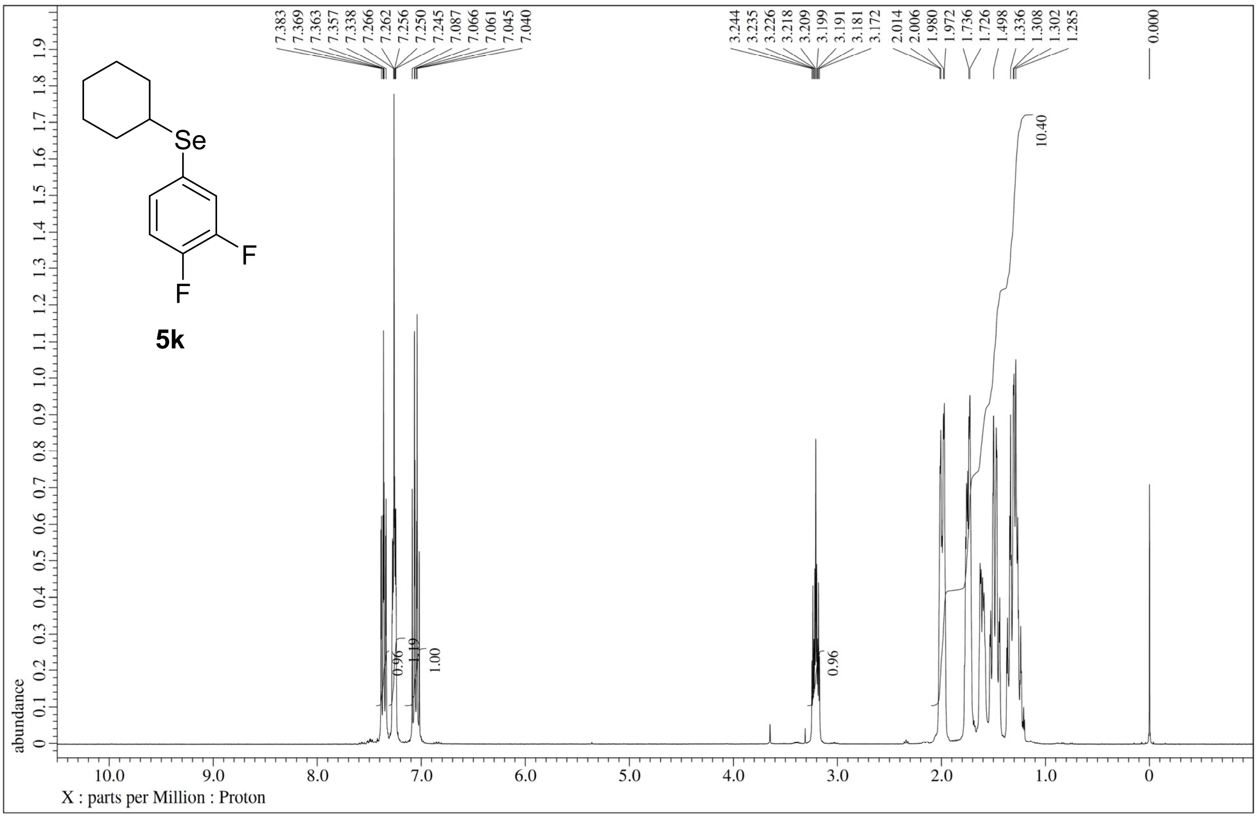
7.383
7.369
7.363
7.357
7.338
7.266
7.262
7.256
7.250
7.245
7.087
7.066
7.061
7.045
7.040
3.244
3.235
3.226
3.218
3.209
3.199
3.191
3.181
3.172
2.014
2.006
1.980
1.972
1.736
1.726
1.498
1.336
1.308
1.302
1.285
0.000
Se
F
F
5k
0.96
1.19
1.00
0.96
10.40
abundance
X : parts per Million : Proton

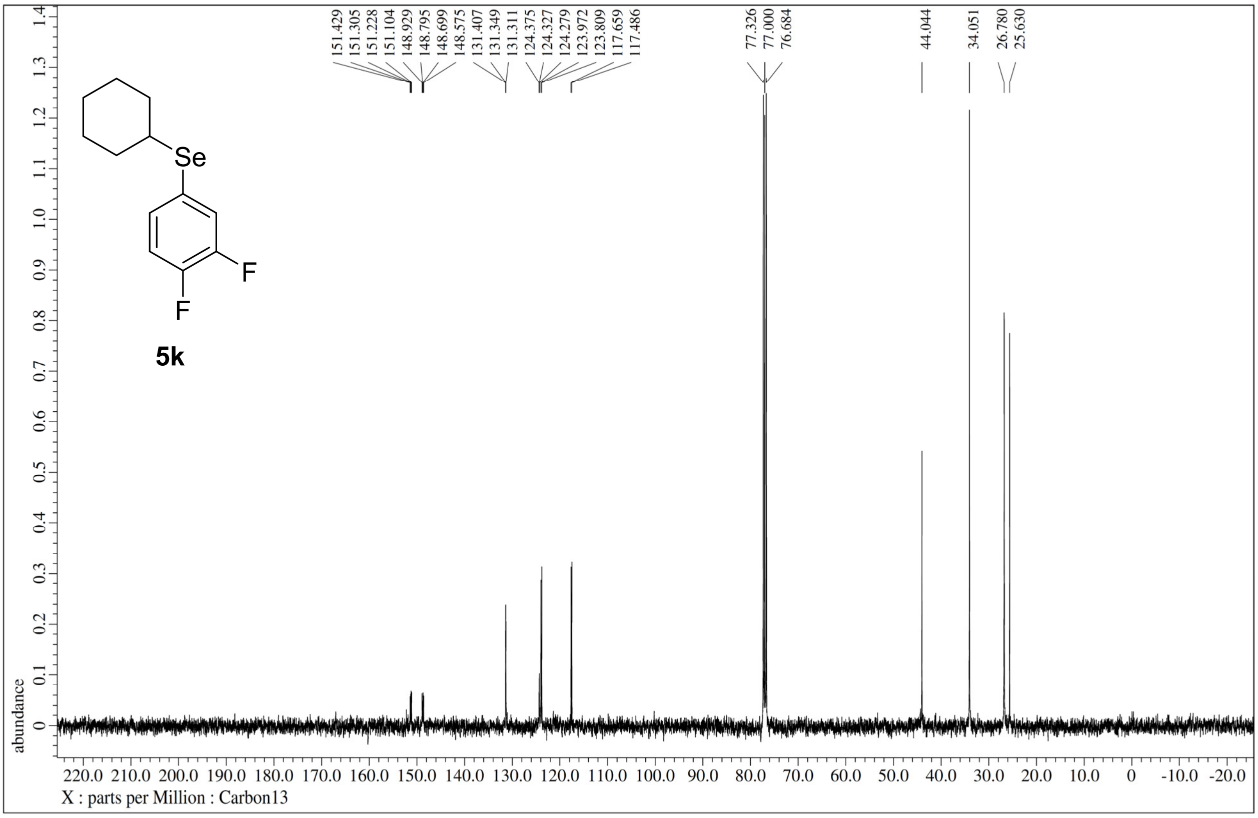
151.429
151.305
151.228
151.104
148.929
148.795
148.699
148.575
131.407
131.349
131.311
124.375
124.327
124.279
123.972
123.809
117.659
117.486
77.326
77.000
76.684
44.044
34.051
26.780
25.630
Se
F
F
5k
abundance
X : parts per Million : Carbon13
